# IRF6 controls Epstein-Barr virus (EBV) lytic reactivation and differentiation in EBV-infected epithelial cells

**DOI:** 10.1371/journal.ppat.1013236

**Published:** 2025-06-26

**Authors:** Stuart A. Fogarty, Deo R. Singh, Scott E. Nelson, Maria E. Calandranis, Yitao Zhang, Abigail S. Pawelski, Alisha S. Kansra, Sophie White, Shannon C. Kenney

**Affiliations:** 1 Department of Oncology, School of Medicine and Public Health, University of Wisconsin-Madison, Madison, Wisconsin, United States of America; 2 Department of Medicine, School of Medicine and Public Health, University of Wisconsin-Madison, Madison, Wisconsin, United States of America; University of Utah, UNITED STATES OF AMERICA

## Abstract

Latent Epstein-Barr virus (EBV) infection promotes undifferentiated nasopharyngeal carcinoma (NPC) and gastric carcinoma (GC), while EBV infection of normal differentiated oropharyngeal epithelial cells is lytic and kills the cell. Establishment of viral latency within epithelial cells is likely essential for the development of EBV-induced NPCs and GCs, but the mechanism(s) by which EBV latency is maintained in epithelial cells are not fully understood. Here we demonstrate that the cellular tumor suppressor protein IRF6, a master regulator of squamous cell epithelial cell differentiation, plays a critical role in promoting TPA-induced lytic EBV reactivation *in vitro* in both EBV-infected NPC cells and EBV-infected GC cells. Using a telomerase-immortalized normal oral keratinocyte cell line (NOKs) model which retains the ability to differentiate in response to TPA treatment, we show that TPA-induced lytic EBV reactivation requires the PKCδ-RIPK4-IRF6 signaling pathway. RIPK4 is a PKCδ (PRKCD)-activated cellular S/T kinase that phosphorylates and activates the IRF6 transcription factor. We demonstrate that inhibition of PKCδ, RIPK4 or IRF6 expression is sufficient to suppress TPA-induced epithelial cell differentiation, as well as lytic EBV reactivation, in NOKs. Furthermore, we find that latent EBV infection in NOKs inhibits the expression of IRF6. Importantly, we show that inducible expression of a constitutively active (phospho-mimetic) IRF6 mutant is sufficient to activate the lytic form of EBV infection in both EBV-infected NOKs and EBV-infected SNU719 GC cells. Finally, we demonstrate that the ability of constitutively active IRF6 to promote lytic EBV infection in NOKs is at least partially mediated by IRF6-induced expression of the BLIMP1 transcription factor, which we previously showed synergistically activates expression of the two EBV immediate-early proteins, BZLF1 and BRLF1, in conjunction with KLF4. Thus, suppression of IRF6 expression may promote NPC and GC tumors by blocking lytic EBV reactivation and differentiation.

## Author summary

EBV is an important cause of human epithelial cell tumors, including undifferentiated nasopharyngeal carcinoma (NPC) and gastric carcinoma (GC). EBV + NPCs and GCs contain latently infected cells that are undifferentiated. The cellular IRF6 transcription factor functions as a tumor suppressor in epithelial cell tumors, including NPC and GC, and promotes differentiation of normal human keratinocytes. Here we show that active IRF6 is required for efficient TPA-induced lytic viral reactivation in EBV-infected normal oral keratinocytes (NOKs) and demonstrate that the lytic-inducing effect of IRF6 in NOKs is closely related to its ability to induce epithelial cell differentiation. Furthermore, we show that expression of constitutively active IRF6 in EBV-infected NOKs and GC cells is sufficient to induce lytic EBV infection. These results suggest that loss of IRF6 expression in EBV-positive NPCs and GCs promotes tumor development by preventing lytic EBV infection and blocking cellular differentiation.

## Introduction

Epstein-Barr virus (EBV) is a gamma herpesvirus that causes infectious mononucleosis and promotes both B cell and epithelial cell malignancies in humans [1]. EBV infection in humans is largely confined to B cells and oropharyngeal epithelial cells. EBV-infected B cells support various different types of latent EBV infection, but can reactivate to the lytic form of viral infection in response to antigen stimulation or plasma cell differentiation [1]. In contrast, EBV infection of normal differentiated oropharyngeal epithelial cells results in lytic EBV infection. Oral hairy leukoplakia (OHL), a non-malignant EBV-induced tongue lesion found in immunosuppressed patients, is caused by lytic EBV infection of differentiated epithelial cells in the absence of latent EBV infection [2,3]. Likewise, *in vitro* EBV infection of organotypic (“rafted”) cultures of primary oral keratinocytes or nasopharyngeal epithelial cells results in lytic viral infection of the differentiated cell layers [4–6]. However, EBV-infected epithelial cell tumors, including nasopharyngeal carcinoma (NPC) and gastric carcinoma (GC), are largely composed of latently infected tumor cells. Although the mechanism(s) by which EBV establishes latency in NPC and GC tumor cells are not yet fully understood, the ability of tumors to achieve viral latency is likely essential for their growth and survival because fully lytic EBV infection kills EBV-infected host cells.

Lytic EBV infection in normal oral epithelial cells is strongly associated with epithelial cell differentiation [1]. Epithelial cell differentiation and loss of cell cycle progression are tightly correlated, and proliferating cells are generally restricted to the undifferentiated basal cell layers In both *in vivo* oral epithelium and *in vitro* rafted normal oral keratinocyte or nasopharyngeal cell models [2–4,7–9]. The cellular Rb tumor suppressor protein has been shown to promote efficient lytic EBV reactivation in differentiated epithelial cells by sequestering E2F transcription factors and inhibiting cell cycle progression [10,11], suggesting that lytic EBV reactivation may be at least partially regulated by cell cycle control mechanism(s) independent of epithelial cell differentiation. In addition, increasing evidence suggests that EBV tightly coordinates its own lytic viral reactivation with epithelial cell differentiation by using the same cellular transcription factors that control normal epithelial cell differentiation to regulate expression of the two EBV immediate-early (IE) proteins, BZLF1 (Z) and BRLF1 (R). Expression of the Z and R EBV IE proteins is required for the activation of the early lytic and late lytic viral proteins that mediate lytic EBV DNA replication and production of infectious viral particles. Thus, cellular transcription factors that regulate Z and/or R expression play essential roles in determining whether EBV infection is latent or lytic in host cells.

Our group previously showed that the cellular transcription factors KLF4 and BLIMP1, which are induced by, and required for, epithelial cell differentiation, promote the lytic form of EBV infection in epithelial cells by activating expression of the Z and R proteins [8,12]. In contrast, we found that the ΔNp63ɑ cellular transcription factor, which is expressed at high levels in undifferentiated basal epithelial cells and lost during epithelial cell differentiation, inhibits expression of the Z and R IE proteins and blocks lytic EBV reactivation [13]. In addition, we recently discovered that activation of the integrated stress response (ISR) pathway induces lytic EBV reactivation in epithelial cells by increasing epithelial cell differentiation and showed that this effect is largely mediated by increased expression of the DDIT3 (CHOP) transcription factor [14].

In this study, we have explored whether EBV lytic reactivation is also regulated by the cellular IRF6 protein, a master regulator of squamous epithelial cell differentiation that controls transcription of other cellular transcription factors that are required for epithelial cell differentiation [15,16]. Mutations in the IRF6 gene are a cause of cleft lip and palate in humans and mice [17,18]. IRF6 regulates the switch between proliferation and differentiation in keratinocytes and the loss of functional IRF6 in mouse skin results in hyperproliferative epidermis that cannot undergo terminal differentiation [19]. IRF6 transcriptionally activates the expression of cellular transcription factors, including KLF4, GRHL3, OVOL1, and IRF6 itself, that mediate keratinocyte differentiation [20–22], while inhibiting expression of ΔNp63ɑ via induction of its proteasomal degradation [23]. The transcriptional activity of IRF6 is controlled by RIPK4-mediated phosphorylation of IRF6 on serine residues 90, 413 and 424, and phosphorylation of residues 413 and 424 is required for nuclear localization of IRF6 [16,24,25]. The ability of TPA treatment (which activates PKC) to induce differentiation of normal human keratinocytes is dependent upon IRF6 activation and involves a TPA-induced signaling pathway in which PKCδ activation leads to RIPK4 auto-phosphorylation/activation, followed by RIPK4-mediated IRF6 phosphorylation and activation [24,26].

IRF6 expression is often silenced by gene methylation in epithelial cell tumors, in which it usually functions as a tumor suppressor [20,25,27–29]. A previous study reported that IRF6 expression is decreased in NPC cells via methylation of the IRF6 promoter and found that overexpression of IRF6 in NPC cells *in vitro* reduces their proliferation [25]. Likewise, IRF6 expression has been reported to be reduced in gastric cancers via DNA methylation of the IRF6 gene promoter, and decreased IRF6 expression in gastric carcinoma correlates with poor clinical prognosis [29]. Interestingly, we recently showed that EBV infection of human telomerase-immortalized normal oral keratinocyte cells (NOKs) inhibits expression of cellular proteins that are activated by epithelial cell differentiation, including IRF6 [30]. We thus hypothesized that loss of IRF6 expression might mediate some of EBV’s effects in NOKs *in vitro* and contribute to EBV-induced NPCs in humans.

Here we have used EBV-infected telomerase-immortalized normal oral keratinocytes (NOKs) as a biologically relevant *in vitro* model for studying whether IRF6 regulates lytic EBV reactivation in epithelial cells and/or contributes to the effects of EBV infection on epithelial cell differentiation. We demonstrate that TPA-induced reactivation of lytic EBV infection in NOKs proceeds through a differentiation-dependent mechanism and show that IRF6 knock-down, as well as knock-down of the upstream IRF6 activators, PKCδ and RIPK4, prevents the TPA-mediated lytic EBV reactivation. Likewise, we find that IRF6 is also required for efficient TPA-induced lytic EBV reactivation in both an EBV + NPC cell line (NPC43) and an EBV+ gastric carcinoma cell line (SNU719). Furthermore, we show that expression of a constitutively active IRF6 mutant (that mimics the effect of RIPK4-mediated IRF6 phosphorylation) is sufficient to induce lytic EBV reactivation in both EBV-infected NOKs and SNU719 cells. Finally, we demonstrate that the ability of constitutively active IRF6 to induce lytic EBV reactivation in NOKs is at least partially mediated by increased expression of the differentiation-dependent transcription factor, BLIMP1, and find that IRF6-regulated expression of the KLF4 and ΔNp63ɑ transcription factors also contributes to lytic EBV reactivation in NOKs. Together, these results suggest that loss of IRF6 expression in EBV-infected epithelial cell tumors promotes tumor formation not only by decreasing epithelial cell differentiation but also by restricting the amount of lytic EBV reactivation.

## Results

### IRF6 is required for efficient TPA-induced lytic EBV reactivation and epithelial cell differentiation in EBV-infected NOKs

**Fig 1:**
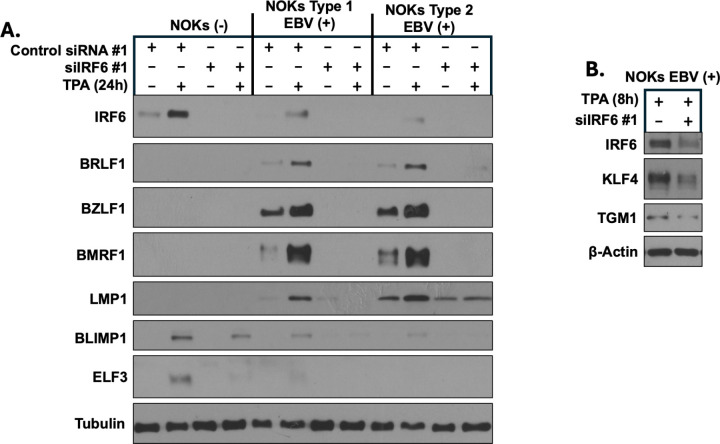
IRF6 expression is required for spontaneous, and TPA-induced, lytic EBV infection and TPA-induced differentiation in NOKs. **(A)** Uninfected NOKs (NOKs (-)) or NOKs infected with AG876 type 2 strain EBV or Akata type 1 strain EBV were plated in the absence of growth factors in KSFM, at a sub-confluent density, treated with a control siRNA or an IRF6-directed siRNA for two days, and then treated with or without TPA for 24 hours before harvesting protein extracts for immunoblot analysis. Expression levels of IRF6, the EBV IE lytic proteins BZLF1 and BRLF1, the early lytic proteins BMRF1 and LMP1, and epithelial differentiation markers BLIMP1 and ELF3, are shown. Tubulin served as a loading control. **(B)** AG876 EBV-infected NOKs were plated in the absence of growth factors in KSFM, at a sub-confluent density, treated with a control siRNA or an IRF6-directed siRNA for two days, and then treated with or without TPA for 8 hours before harvesting protein extracts for immunoblot analysis. Expression levels of IRF6 and the differentiation markers, KLF4 and TGM1, are shown. Actin served as a loading control.

The phorbol ester 12-O-Tetradecanoylphorbol-13-acetate (TPA) activates PKC and is a well-known inducer of lytic EBV reactivation. We have previously shown that EBV-infected and uninfected NOKs can undergo some degree of “spontaneous” differentiation and lytic EBV reactivation when cells are grown in sub-confluent conditions in the absence of growth factors and demonstrated that TPA treatment further enhances NOKs differentiation and lytic EBV reactivation [14]. To determine if IRF6 expression is required for the ability of TPA to induce lytic EBV reactivation in NOKs, uninfected NOKs, or NOKs infected with either a type 1 EBV (Akata) strain or a type 2 EBV (AG876) strain were treated with a control siRNA or an IRF6-targeted siRNA for two days and then treated with TPA (or DMSO solvent) for an additional day. Protein extracts were then harvested and immunoblot analysis performed to examine the amount of lytic EBV protein expression activated by TPA treatment. As shown in Fig 1A, TPA treatment increased IRF6 expression in both uninfected and EBV-infected NOKs, and greatly enhanced expression of EBV lytic proteins, including the immediate-early proteins BZLF1 and BRLF1, and the early lytic proteins BMRF1 and LMP1; LMP1 can be expressed at low levels as a latent protein in NOKs and at higher levels as a lytic protein [14]. Importantly, pretreatment of cells with an IRF6-targeted siRNA inhibited both TPA-induced and spontaneous lytic EBV reactivation. Similar results were obtained in NOKs using a second control siRNA and a second IRF6-directed siRNA (S1 Fig). Of note, the EBV-infected NOKs express considerably less IRF6 protein relative to that expressed in uninfected NOKs (Figs 1A and S1), consistent with our previous findings showing that EBV infection inhibits NOKs differentiation [30,31]. These results reveal that IRF6 expression is induced by TPA treatment in NOKs and show that IRF6 activation is indeed required for efficient TPA-mediated as well as spontaneous lytic EBV reactivation in NOKs.

As TPA treatment is known to induce keratinocyte differentiation via its effects on PKC and is commonly used to study differentiation in normal keratinocytes [24,32], we also examined whether knock-down of IRF6 expression in NOKs is associated with a decrease in TPA-mediated epithelial cell differentiation. As shown in Fig 1A, TPA treatment increased the expression levels of the differentiation-induced proteins, BLIMP1 and ELF3, although, as expected, this differentiating effect was more marked in the uninfected cells. Importantly, knock-down of IRF6 expression greatly decreased the TPA-induced expression of differentiation-indued cellular proteins, BLIMP1, ELF3, KLF4 and TGM1 (Fig 1A and 1B). The siIRF6 effect on KLF4 expression was studied after 8 hours of TPA treatment (Fig 1B) because we found that KLF4 expression was lost after 24 hours of TPA treatment. Similar results were obtained using a second siRNA directed against IRF6 (S1 Fig). Of note, EBV-infected NOKs treated with the combination of TPA and siIRF6 did not show obvious toxicity or reduced proliferation in comparison to the untreated cells (S2 Fig). These results suggest that IRF6 may promote TPA-induced lytic EBV reactivation in NOKs by activating TPA-mediated epithelial cell differentiation.

### IRF6 is also required for efficient TPA-induced lytic EBV reactivation in EBV-infected NPC43 NPC cells and EBV-infected SNU719 gastric carcinoma cells

**Fig 2:**
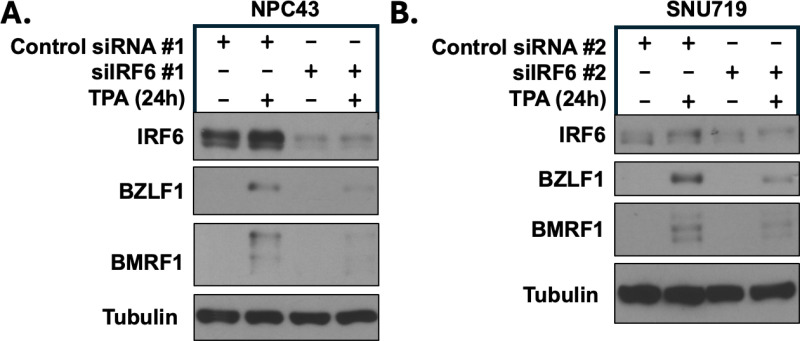
IRF6 expression is required for TPA-induced lytic EBV infection in EBV-infected nasopharyngeal carcinoma NPC43 and gastric carcinoma SNU719 cells. **(A)** NPC43 cells were treated with a control siRNA or an IRF6-directed siRNA for 24 hours, then treated with or without TPA for 24 hours before harvesting protein extracts for immunoblot analysis. Expression levels of IRF6 and the EBV lytic proteins BZLF1 and BMRF1 are shown. Tubulin served as a loading control. **(B)** SNU719 cells were treated with a control siRNA or an IRF6-directed siRNA for 24 hours, then treated with or without TPA for 24 hours before harvesting protein extracts for immunoblot analysis. Expression levels of IRF6 and the EBV lytic proteins BZLF1 and BMRF1 are shown. Tubulin served as a loading control.

To determine if the requirement for IRF6 for efficient TPA-mediated lytic EBV reactivation is specific to NOKs, we next examined the effect of IRF6 knock-down on TPA-induced lytic EBV reactivation in an EBV + NPC cell line, NPC43, and an EBV + GC cell line, SNU719. Importantly, both the NPC43 and SNU719 cell lines were obtained from EBV+ patient tumors rather than from *in vitro* EBV “super-infection” of uninfected NPC and gastric tumors [33,34]. As shown in Figs 2 and S3, knock-down of IRF6 expression using two different IRF6 siRNAs decreased the amount of TPA-induced lytic EBV reactivation in both cell lines. Interestingly, the level of constitutive IRF6 expression in NPC43 cells was relatively high (similar to uninfected NOKs), likely reflecting the recently described “hypomethylated” state of this cell line in contrast to most NPC tumors which have a “hypermethylated” phenotype [35]. Nevertheless, IRF6 knock-down decreased lytic EBV reactivation in TPA-treated NPC43 cells.

### TPA-mediated lytic EBV reactivation in NOKs also requires PKCδ and RIPK4 expression

**Fig 3:**
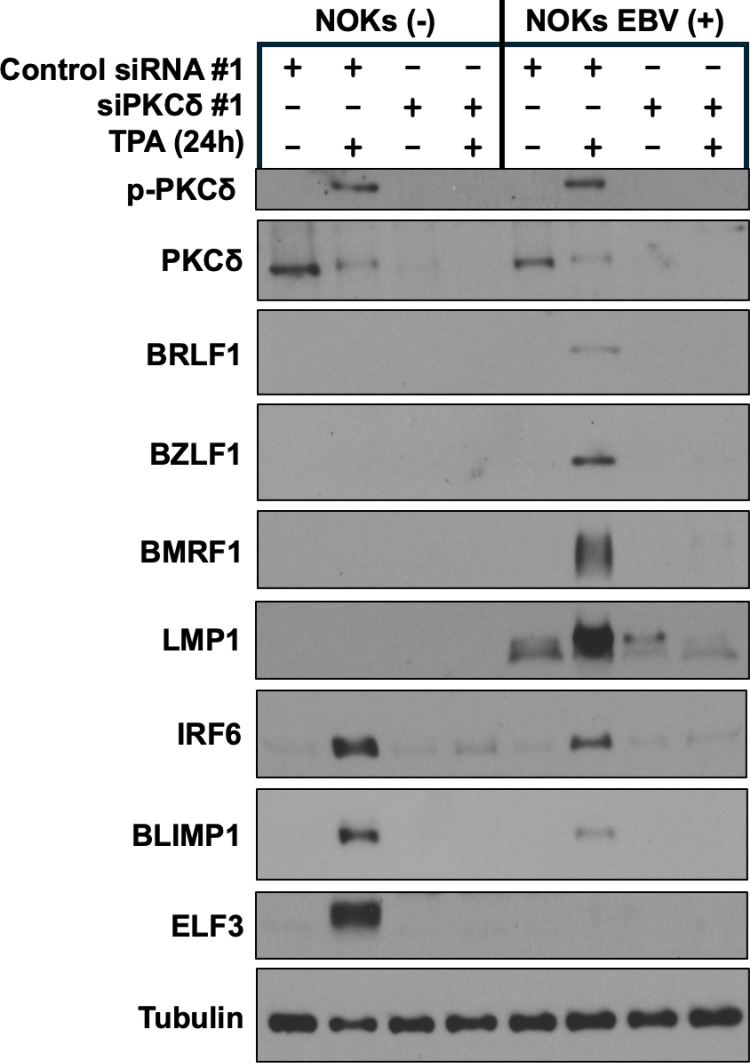
PKCδ expression is required for TPA-induced lytic EBV infection and TPA-induced differentiation in NOKs. Uninfected NOKs (NOKs (-)) or NOKs infected with AG876 type 2 strain EBV (NOKs EBV(+)) were plated in the absence of growth factors in KSFM, at a sub-confluent density, treated with a control siRNA or a PKCδ-directed siRNA for two days, and then treated with or without TPA for 24 hours before harvesting protein extracts for immunoblot analysis. Expression levels of PKCδ, the EBV lytic proteins BRLF1, BZLF1, BMRF1, and LMP1, and the differentiation markers IRF6, BLIMP1, and ELF3 are shown. Tubulin served as a loading control.

As TPA-mediated activation of IRF6 in keratinocytes has been previously shown to require both PKC activation and RIPK4 activation [24,26], we next determined whether knock-down of either PKCδ or RIPK4 expression also prevents TPA-mediated lytic EBV reactivation and/or epithelial cells differentiation in EBV-infected NOKs. We chose to knock-down the PKCδ form of PKC as PKCδ has been previously shown to be required for keratinocyte differentiation [36]. As shown in Fig 3, as expected, TPA treatment of uninfected and EBV-infected cells induced phosphorylation (and activation) of PKCδ and destabilized it [37]. Furthermore, knock-down of PKCδ dramatically decreased TPA-mediated induction of lytic EBV proteins (including BRLF1, BZLF1, BMRF1 and LMP1). Knock-down of PKCδ also blocked TPA-mediated expression of epithelial cell differentiation markers, including IRF6, BLIMP1 and ELF3 (Fig 3). Similar results were obtained using a second siRNA directed against PKCδ (S4 Fig). These results confirm that PKCδ is required for both efficient TPA-mediated lytic EBV reactivation and efficient TPA-mediated differentiation in NOKs.

**Fig 4:**
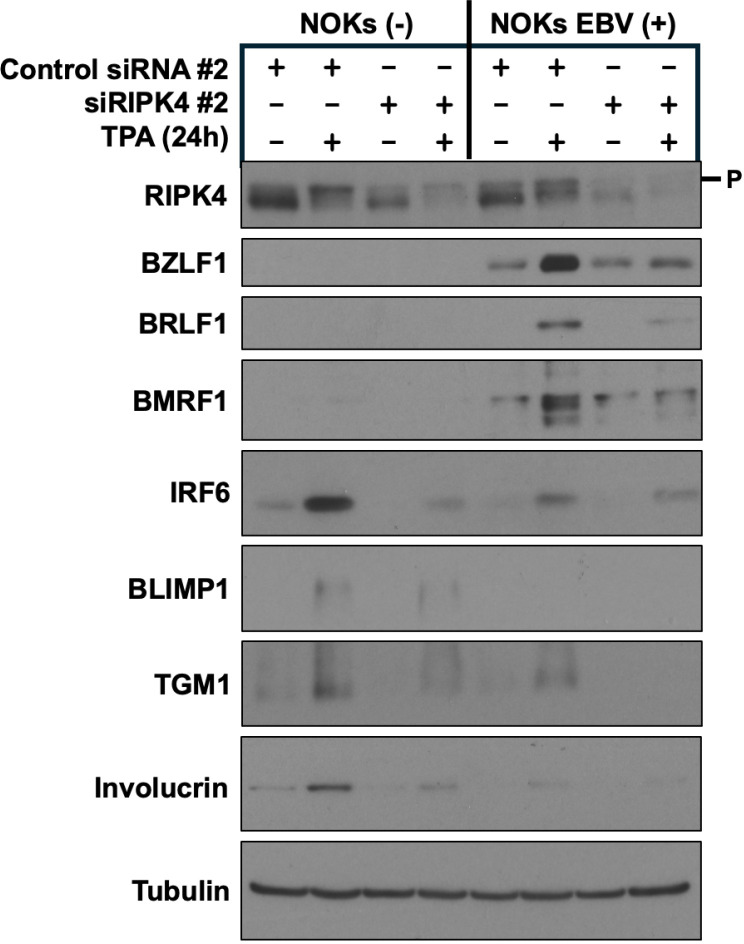
RIPK4 expression is required for TPA-induced lytic EBV infection and TPA-induced differentiation in NOKs. Uninfected NOKs (NOKs (-)) or NOKs infected with AG876 type 2 strain EBV (NOKs EBV(+)) were plated in the absence of growth factors in KSFM, at a sub-confluent density, treated with a control siRNA or a RIPK4-directed siRNA for two days, and then treated with or without TPA for 24 hours before harvesting protein extracts for immunoblot analysis. Expression levels of RIPK4, the EBV lytic proteins BRLF1, BZLF1, and BMRF1, and the differentiation markers IRF6, BLIMP1, TGM1, and involucrin are shown. Tubulin served as a loading control. The size of phosphorylated RIPK4 is indicated by “P”.

We next asked if knock-down of RIPK4 attenuates TPA-induced lytic EBV reactivation in NOKs. We found that TPA treatment induces phosphorylation of RIPK4 and decreases its stability (Fig 4), as previously described [26]. Importantly, knock-down of RIPK4 greatly inhibited TPA-induced lytic EBV protein expression. as well as expression of TPA-activated differentiation proteins, IRF6, BLIMP1, TGM1 and involucrin (Fig 4). Similar results were obtained using a second siRNA directed against RIPK4 (S5 Fig). Together, these results indicate that both TPA-induced lytic EBV reactivation and TPA-mediated epithelial cell differentiation proceed through a PKCδ-RIPK4-IRF6 signaling pathway in NOKs.

### Expression of constitutively active IRF6 is sufficient to induce lytic EBV reactivation and epithelial cell differentiation in EBV-infected NOKs

**Fig 5:**
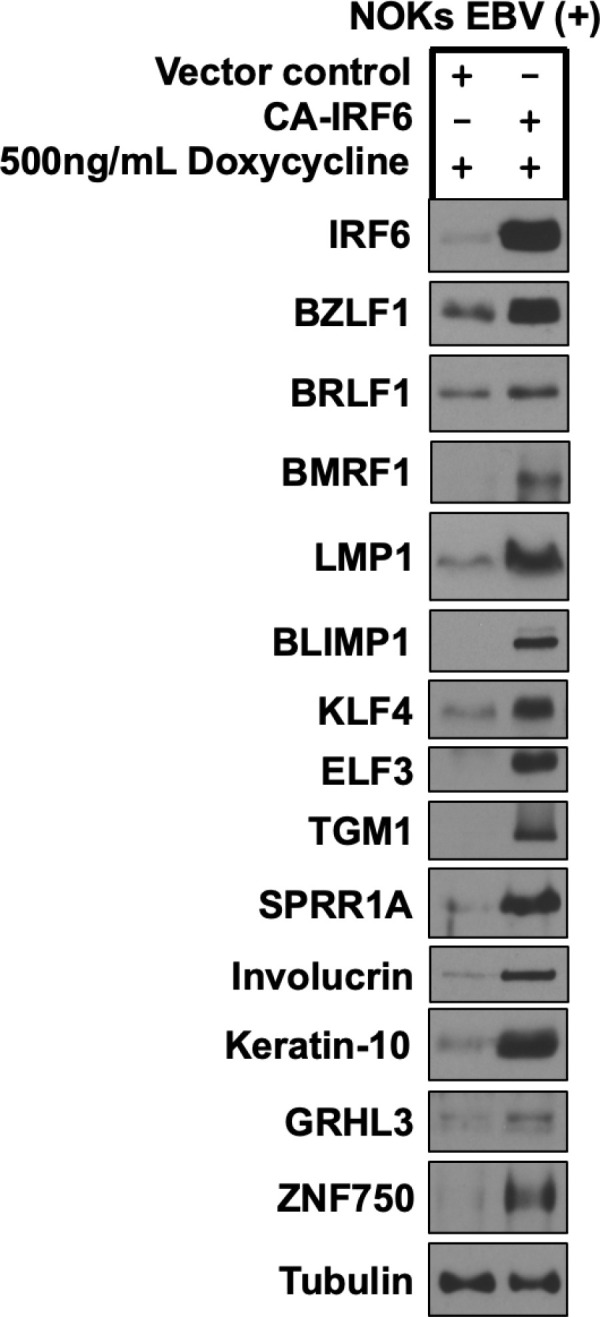
Constitutively active IRF6 induces lytic EBV reactivation and epithelial cell differentiation in EBV-infected NOKs. AG876 type 2 strain EBV-infected NOKs were stably infected with a control vector or a lentivirus expressing a doxycycline inducible phospho-mimetic IRF6 mutant (CA-IRF6, in which serine residues 413 and 424 were switched to glutamic acid), selected with puromycin for five days, and then treated with 500ng/mL doxycycline for 72 hours and examined by immunoblot analyses to examine expression of IRF6, lytic EBV proteins BZLF1, BRLF1, BMRF1, and LMP1, and the differentiation markers BLIMP1, KLF4, ELF3, TGM1, SPRR1A, Involucrin, Keratin 10, GRHL3, and ZNF750 as shown. Tubulin served as a loading control.

**Fig 6:**
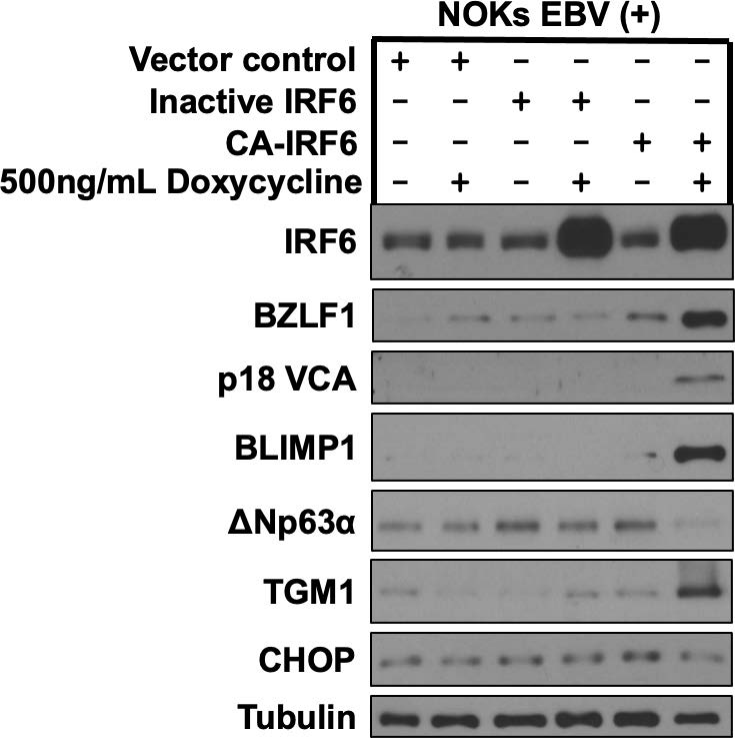
An inactive IRF6 mutant does not induce lytic EBV reactivation and epithelial cell differentiation in EBV-infected NOKs. Akata type 1 strain EBV-infected NOKs were stably infected with a control vector or a lentivirus expressing a doxycycline inducible phospho-mimetic IRF6 mutant (CA-IRF6, in which serine residues 413 and 424 were switched to glutamic acid) or an inactive IRF6 mutant (Inactive IRF6, in which serine residues 413 and 424 were switched to alanine), then treated with 500ng/mL doxycycline for 72 hours and examined by immunoblot analyses to examine expression of IRF6, the IE lytic EBV protein BZLF1 and the late lytic EBV protein p18 VCA, the differentiation markers BLIMP1, ΔNp63α, and TGM1, and the integrated stress response protein CHOP as shown. Tubulin served as a loading control.

IRF6 activity is enhanced during epithelial differentiation both by increased transcription of the IRF6 gene (mediated by IRF6 activation of its own promoter) and increased RIPK4-mediated phosphorylation of the IRF6 protein on serine residues 413 and 424. To mimic the effects of RIPK4-mediated phosphorylation, versus unphosphorylated IRF6, we constructed doxycycline-inducible IRF6 expressing lentivirus vectors in which IRF6 serine residues 413 and 424 are converted to either glutamic acid residues (constitutively active) or alanine residues (inactive) as described in the methods section. When EBV-infected NOKs were infected with control or constitutively active IRF6-expressing lentivirus vectors and then treated with or without doxycycline for 3 days to induce expression of the constitutively active IRF6 protein, we found that the constitutively active (phospho-mimetic) IRF6 protein efficiently induced expression of lytic EBV proteins, including the two EBV IE proteins BZLF1 and BRLF1, and the early lytic EBV proteins, BMRF1 and LMP1, in the absence of TPA treatment (Fig 5). Furthermore, expression of constitutively active IRF6 was also sufficient to induce expression of many differentiation-dependent cellular proteins, including BLIMP1, KLF4, ELF3, TGM1, SPRR1A, involucrin, K10, GRHL3 and ZNF750) in the absence of TPA treatment (Fig 5). However, constitutively active IRF6 did not induce expression of the ISR activated protein CHOP (S6 Fig), suggesting that CHOP activation is upstream of IRF6 activation in TPA-treated NOKs. Similar results were obtained in another experiment (S7 Fig). Constitutively active IRF6 also decreased expression of ΔNp63ɑ in this experiment (S7 Fig), a transcription factor expressed in basal epithelial cells that potently decreases lytic EBV reactivation in NOKs [13],

The ability of constitutively active IRF6 to induce lytic EBV reactivation and epithelial cell differentiation in NOKs was not associated with any obvious cellular toxicity (S8 Fig) and did not occur when the IRF6 serine residues 413 and 424 were both converted to alanine residues instead of glutamic acid residues (Fig 6). As expected, IHC staining of EBV-positive NOKs cells infected with the constitutively active IRF6 protein showed an increased number of IRF6- and Z- expressing cells (S9 Fig). These results suggest that the lytic inducing effect of wild-type IRF6 requires RIPK4-mediated phosphorylation of serine residues 413 and/or 424 and is not due to some non-specific toxicity of IRF6 over-expression. Thus, the phospho-mimetic IRF6 mutant mimics the effect of TPA treatment in NOKs and bypasses the requirement for PKCδ and RIPK4 activity.

### Expression of constitutively active IRF6 induces lytic EBV reactivation in EBV-infected SNU719 GC cells

**Fig 7:**
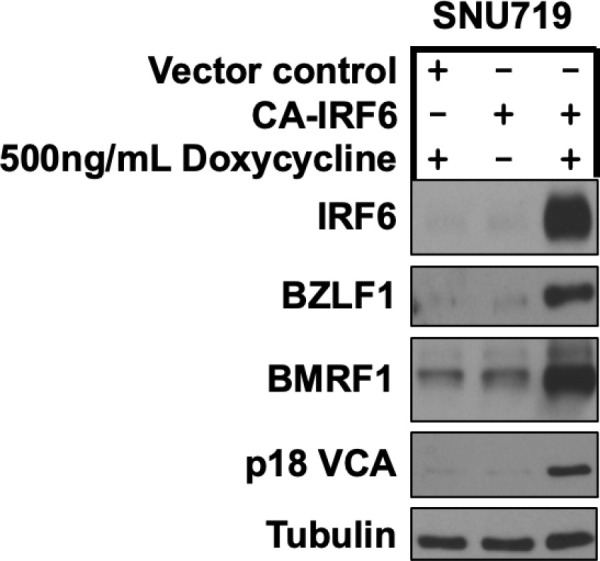
Constitutively active IRF6 induces lytic EBV reactivation in EBV-infected SNU719 gastric carcinoma cells. EBV-infected SNU719 cells were stably infected with a control vector or a lentivirus expressing a doxycycline inducible phospho-mimetic IRF6 mutant (CA-IRF6, in which serine residues 413 and 424 were switched to glutamic acid) then treated with 500ng/mL doxycycline for 72 hours and examined by immunoblot analyses to examine expression of IRF6 and IE lytic EBV protein BZLF1, the early lytic EBV protein BMRF1, and the late lytic EBV protein p18 VCA as shown. Tubulin served as a loading control.

To determine if constitutively active IRF6 expression also induces lytic EBV reactivation in EBV-infected GC cells, SNU719 cells were infected with control lentivirus vector or the lentivirus vector expressing constitutively active IRF6, selected with puromycin treatment for several days, and then treated with or without doxycycline to induce IRF6 expression. As shown in Fig 7, constitutively active IRF6 expression induced expression of the EBV lytic viral proteins, Z, BMRF1 and p18 VCA in SNU719 cells. Similar results were observed in a second experiment (S10 Fig). Similar to its effect in EBV-infected NOKs, the constitutively active IRF6 mutant also increased expression of the BLIMP1 and KLF4 transcription factors in SNU719 cells (S10 Fig), although this effect was not as strong as that observed in NOKs.

### Both BLIMP1 and KLF4 expression are required for IRF6-induced lytic EBV reactivation in NOKs

**Fig 8:**
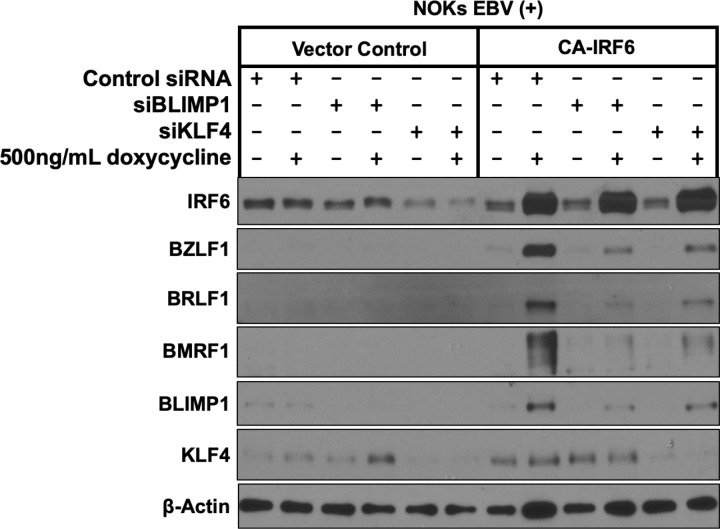
BLIMP1 and KLF4 expression are required for CA-IRF6-induced lytic EBV reactivation in NOKs. NOKs infected with type 1 strain Akata EBV (NOKs EBV(+)) were stably infected with a control vector or a lentivirus expressing a doxycycline inducible phospho-mimetic IRF6 mutant (CA-IRF6) and then transfected with control siRNA or siRNAs directed to KLF4 or BLIMP1 as indicated. 24h following transfection, cells were treated with 500ng/ml doxycycline for 72 hours and immunoblot analyses were performed to examine expression of CA-IRF6, BLIMP1, KLF4, and lytic EBV proteins BZLF1, BRLF1 and BMRF1 as shown. Actin serves as a loading control.

We previously showed that expression of the differentiation-induced cellular transcription factors, KLF4 and BLIMP1, synergistically induces lytic EBV reactivation in NOKs by activating the BZLF1 and BRLF1 IE promoters [8]. We therefore hypothesized that the ability of constitutively active IRF6 to induce lytic EBV reactivation in NOKs might be mediated indirectly via activation of the BLIMP1 and/or KLF4 transcription factors. To determine if BLIMP1 and/or KLF4 expression is required for the ability of constitutively active IRF6 to induce lytic EBV reactivation in NOKs, EBV-positive NOKs (infected with the doxycycline-inducible IRF6 vector) were treated for one day with control siRNA or siRNAs targeting KLF4 or BLIMP1, followed by two days of doxycycline treatment to induce IRF6 expression. Immunoblot analysis was then performed to examine the effect of each treatment on expression of lytic EBV proteins and cellular differentiation markers. As shown in Fig 8, expression of constitutively active IRF6 in EBV-infected NOKs increased BLIMP1 expression, although it did not increase KLF4 expression in this particular experiment. Knock-down of either IRF6-induced BLIMP1 or constitutive KLF4 expression greatly decreased IRF6-mediated induction of the EBV lytic proteins, Z, R and BMRF1. Similar results were observed in a second experiment using different BLIMP1 and KLF4 siRNAs (S11 Fig). These results suggest that IRF6-induced lytic EBV reactivation in EBV-infected NOKs is at least partially mediated indirectly through activation of the BLIMP1 transcription factor and requires expression of the KLF4 transcription factor as well.

### Expression of the EBV IE proteins, BZLF1 and BRLF1, reverses the inhibitory effect of IRF6 knock-down on lytic EBV reactivation in TPA-treated EBV + NOKs

Given the finding above showing that IRF6 mediated lytic reactivation in NOKs requires KLF4 and BLIMP1, both of which have been shown to activate the EBV IE BZLF1 and BRLF1 promoters, we hypothesized that the ability of IRF6 to induce lytic EBV reactivation is mediated by enhanced activity of the EBV IE promoters. Nevertheless, as it is possible that IRF6 also influences the transcriptional functions of the BZLF1 and/or BRLF1 IE proteins, we asked whether restoration of BZLF1 and BRLF1 protein expression (using doxycycline-inducible BZLF1 and BRLF1 lentivirus vectors) can reverse the effect of IRF6 knock-down in TPA-treated EBV-infected NOKs. As shown in S12 Fig, doxycycline-induced expression of the BZLF1 and BRLF1 EBV IE proteins (in EBV + NOKs infected with lentivirus vectors expressing doxycyline inducible Z and R proteins) restored expression of TPA-induced early lytic EBV protein BMRF1 in TPA-treated cells when IRF6 expression was knocked down. These results suggest that the major effect of IRF6 on lytic EBV reactivation in EBV-infected epithelial cells is due to up-regulated expression of the EBV IE genes.

## Discussion

EBV infection contributes to the formation of undifferentiated NPCs, and the EBV genome is already present within the earliest premalignant NPC lesions [38]. The two viral latent membrane proteins, LMP1 and LMP2A, along with the virally encoded microRNAs, are thought to be the major EBV-dependent drivers of NPC [1]. However, the ability of EBV to promote early NPC lesions also requires that it successfully establish persistent viral latency in a cell type (epithelial cells) that often supports completely lytic infection [4,7]. Mounting evidence from our lab and others suggests that differentiation of epithelial cells reactivates the lytic form of EBV infection. Thus, the establishment of EBV latency in developing NPC tumors may require that the tumor cells be unable to differentiate. Here we have discovered that activation of a master regulator of squamous epithelial cell differentiation, IRF6, is required and sufficient to induce the lytic form of EBV infection and cellular differentiation in normal telomerase-immortalized oral keratinocytes (NOKs). Furthermore, we show that latent EBV infection in NOKs suppresses IRF6 expression, resulting in both decreased cellular differentiation and attenuation of lytic reactivation. Inhibition of IRF6 expression may thus be an important mechanism by which EBV infection promotes early NPC tumors.

We previously demonstrated that latent EBV infection inhibits epithelial cell differentiation in NOKs induced by a variety of different stimuli, including culturing cells on organotypic raft cultures, suspending cells in methylcellulose, and spontaneous differentiation that occurs when cells are grown in the absence of growth factors [9,30,31]. Using an LMP1-deleted EBV mutant, we found that LMP1 expression is required, and sufficient, for the ability of EBV to efficiently inhibit spontaneous differentiation that occurs under growth factor deficient conditions [30]. We also showed that LMP1-mediated inhibition of the Hippo signaling pathway, and subsequent activation of the YAP and TAZ transcription factors, is at least partially responsible for the reduced differentiation in EBV-infected NOKs [30]. In addition, we recently discovered that LMP1-mediated inhibition of the cellular Integrated Stress Response (ISR) pathway contributes to the ability of LMP1 to inhibit epithelial cell differentiation and lytic EBV reactivation, and found that ISR activation of CHOP (DDIT3) expression is sufficient to induce both lytic EBV reactivation and differentiation in NOKs [14].

IRF6 expression is reported to be decreased in NPC tumors *in vivo* relative to its expression in nearby normal epithelial cells, suggesting that loss of IRF6 often occurs in EBV-infected NPCs [25]. Although we previously showed that two other differentiation-induced transcription factors, KLF4 and BLIMP1, can synergistically induce lytic EBV reactivation in epithelial cells by activating the BZLF1 and BRLF1 IE promoters [8], the role of IRF6 in lytic EBV reactivation in epithelial cells has not been previously examined. We hypothesized that IRF6 is a critical regulator of lytic EBV reactivation in epithelial cells, as other investigators have shown that IRF6 functions as the “master regulator” of epithelial cell differentiation, with its activation likely occurring upstream of KLF4 and BLIMP1 expression [15,16]. In addition, the ability of TPA to induce differentiation in normal human keratinocytes has been shown to require IRF6 activation through a PKCδ-RIPK4-IRF6 signaling pathway [24]. Our studies here are the first to reveal that IRF6 activation is indeed essential, and sufficient, to induce lytic viral reactivation in EBV-infected NOKs. Furthermore, our demonstration that knock-down of constitutive IRF6 expression inhibits TPA-induced lytic EBV infection in both an EBV + NPC cell line (NPC43) and an EBV + GC cell line (SNU719) suggests that IRF6 also plays a role regulating the latent-to-lytic EBV switch in human NPC and GC tumors.

Our findings here show that IRF6 expression is required both for the spontaneous lytic EBV reactivation in NOKs that occurs when cells are grown in the absence of growth factors, and for TPA-induced lytic EBV reactivation and differentiation. Furthermore, our experiments reveal that both PKCδ and RIPK4 expression are also required for lytic EBV reactivation and cellular differentiation in response to TPA treatment. Our findings suggest that RIPK4-mediated phosphorylation of IRF6 plays an essential role in TPA-mediated cellular differentiation and lytic EBV reactivation in NOKs, as expression of a phospho-mimetic IRF6 mutant was found to induce both lytic EBV reactivation and cellular differentiation even in the absence of TPA treatment. We also found that both BLIMP1 and KLF4 expression are necessary for IRF6-mediated lytic EBV reactivation and differentiation. These results suggest that the activated (phosphorylated) form of IRF6 induces lytic EBV reactivation in NOKs by initiating epithelial cell differentiation, thereby enhancing expression and/or activities of downstream transcription factors such as BLIMP1 and KLF4 that activate the EBV IE promoters, and inhibiting expression of ΔNp63ɑ, a transcription factor that decreases expression of the EBV IE proteins. Of note, our finding that both PKCδ and RIPK4 expression are also required (upstream of IRF6) for lytic EBV reactivation in NOKs suggests that pharmacologic inhibitors of either PKCδ and/or RIPK4 might be useful for preventing lytic EBV infection in epithelial cells. Interestingly, however, in this study we did not find that constitutively active IRF6 expression in NOKs is sufficient to induce expression of the ISR-activated DDIT3 (CHOP) transcription factor (which can also activate lytic EBV reactivation and epithelial cell differentiation), whereas we previously found that CHOP expression is sufficient to induce IRF6 expression in NOKs [14]. These results suggest that CHOP activation is upstream of IRF6 activation, although the exact mechanism(s) by which CHOP induces IRF6 activation are currently not known.

**Fig 9:**
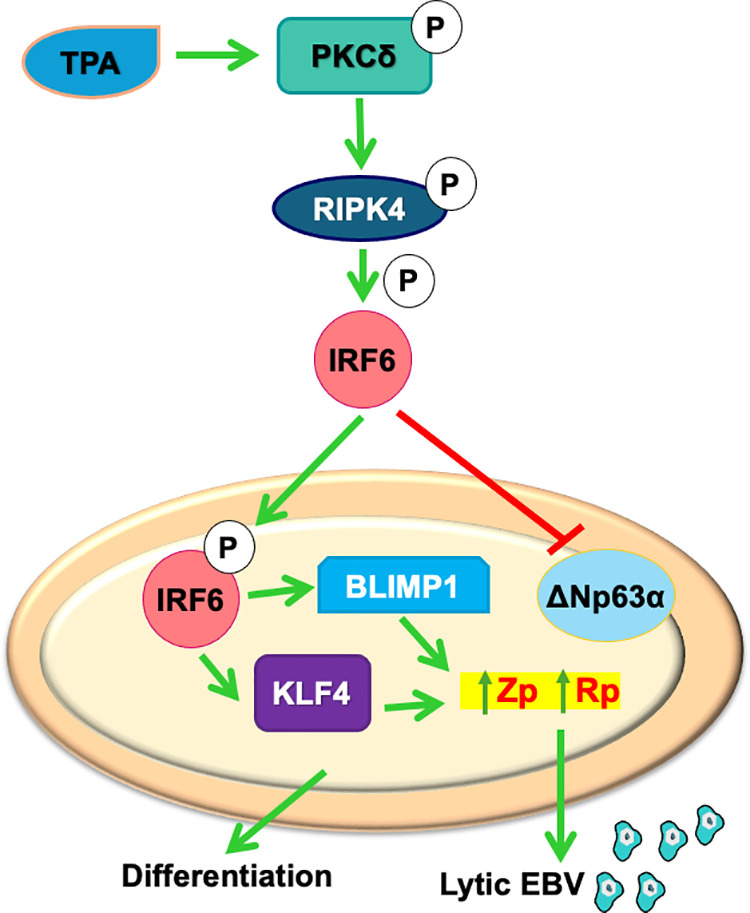
Model of TPA-induced activation of a PKCδ/RIPK4/IRF6 signaling pathway inducing lytic EBV reactivation and epithelial cell differentiation in EBV-infected NOKs.

A model for how TPA treatment induces both epithelial cell differentiation and lytic EBV reactivation in NOKs via activation of IRF6 is shown in Fig 9. This proposed model is likely to be also applicable to the effects of IRF6 on lytic EBV reactivation and cellular differentiation in EBV-infected NPC tumors, as we found that IRF6 knock-down inhibits the ability of TPA to induce lytic EBV reactivation in the NPC cell line, NPC43, *in vitro*. Interestingly, our results here also clearly indicate that IRF6 promotes lytic EBV reactivation in the GC cell line SNU719, as we found that knock-down of IRF6 expression inhibits TPA-induced lytic EBV reactivation and expression of constitutively active IRF6 is sufficient to activate lytic EBV reactivation in this cell type. Our results in SN719 cells suggest that IRF6-induced lytic EBV reactivation in GC cells, similar to its effect in NOKs, may also be mediated by enhanced expression of BLIMP1 and KLF4 (S10 Fig). Nevertheless, as relatively little is currently known about the effects of BLIMP1 and KLF4 expression on normal gastric cell and GC tumor cell differentiation, the ability of IRF6 to induce lytic EBV reactivation in GC tumors may also be mediated via additional (BLIMP1/KLF4-independent) mechanisms.

In addition to its effects on epithelial cell differentiation, recent studies suggest that IRF6 may function as a tumor suppressor for various types of human epithelial cell tumors through a variety of additional mechanisms. For example, when over-expressed in NPC cell lines *in vitro,* IRF6 was found to suppress tumor cell proliferation and the cancer stem cell phenotype, and to increase the sensitivity of cells to chemotherapy treatment [25]. Like NPC, IRF6 expression is also commonly decreased in human gastric cancer, and IRF6 expression in gastric carcinomas is correlated with better patient survival [29]. IRF6 also acts as a tumor suppressor in human squamous cell carcinomas, in which its expression is commonly inhibited [20]. Furthermore, knock-down of IRF6 in primary human keratinocytes *in vitro* not only dysregulates differentiation-induced genes and cell cycle-related genes, but also genes regulating cell-cell contact and cell adhesion and increases the invasive behavior of the cells [20].

Importantly, IRF6 expression in malignant cells also enhances the host immune response to tumor cells. In a mouse model for pancreatic ductal adenocarcinoma, acquired resistance of tumor cells to immune checkpoint inhibition was shown to be mediated by decreased expression of IRF6, which results in an inability of tumor cells to be killed by TNF-α released from immune cells [39]. In addition, a variant in the 3’ UTR of IRF6 that decreases its expression was shown to be associated with decreased survival and enhanced M2 macrophage infiltration of colorectal tumors in humans, and IRF6 was found to promote M2 macrophage polarization *in vitro* [40]. Interestingly, HPV 16, like EBV, has also been reported to inhibit expression of IRF6 in HPV-infected keratinocytes, and this effect (mediated by the HPV E6 oncoprotein) was found to inhibit expression of the IL-1β cytokine [41]. Thus, down-regulation of IRF6 expression may enhance immune evasion in both virally-infected and malignant epithelial cells.

In summary, our results here reveal that activation of the IRF6 transcription factor is critical for lytic EBV reactivation and cellular differentiation in EBV-infected NOKs and suggest that the ability of latent EBV infection to inhibit IRF6 expression in epithelial cells is an important mechanism by which EBV promotes the early development of undifferentiated NPC and GC in humans.

## Materials and methods

### Cell culture

Normal Oral Keratinocytes (NOKs) cells (a gift from Karl Munger of Tufts University via Paul Lambert of the University of Wisconsin) were cultured in Gibco KSFM. To grow cells, 500 ml of media was supplemented with 12.5mg of bovine pituitary extract (BPE), 0.1μg of epidermal growth factor (EGF) and 1% penicillin/streptomycin. For differentiation and lytic reactivation experiments, the BPE and EGF were removed from the media. The generation of Akata (type 1 strain) and AG876 (type 2 strain) EBV-infected NOKs was described previously [31]. EBV-infected NOKs were kept in the same media as the uninfected NOKs except for the presence of 50μg/mL G418. NPC43 cells (a gift from Sai Wah Tsao at the University of Hong Kong) were derived from is a nasopharyngeal carcinoma as described previously [42] and were cultured in Gibco RPMI 1640 supplemented with 10% FBS, 1% penicillin/streptomycin and 4 µM ROCK inhibitor (Y-27632)(CAYMAN). LentiX HEK293 cells (obtained from ATCC) were cultured in RPMI 1640 supplemented with 10% FBS, 1% NEAA, 1% Na Pyruvate, and 1% penicillin/streptomycin. SNU719 cells [34,43] were derived from an EBV+ gastric carcinoma as described previously and were a kind gift of Kenzo Takada, Hokkaido University, Japan (via the Bill Sugden lab). SNU719 cells were cultured in Gibco RPMI 1640 supplemented with 10% FBS, and 1% penicillin/streptomycin.

### Plasmids

Plasmid DNA was prepared using the Qiagen Midi-prep kit according to the manufacturer’s instructions. The plasmid pCW (Addgene Catalog number 184708, a gift from Alessia Ciarrocchi & Gloria Manzotti) was purchased from Addgene and the plasmid pLenti6.3 IRF6 (Catalog Number HsCD00860693) was purchased from DNASU (Tempe, AZ). The IRF6 gene was then modified to produce a constitutively active form by PCR amplification to insert glutamic acid in place of the serine residues at amino acid 413 and 424. The PCR product was then ligated using the T4 DNA ligase. The following primers were used to create the constitutively active IRF6 (CA-IRF6):

FW: 5’-GTCCGCCTGCAGATGAAACCCCAGACATCAAGGATA-3’

RV: 5’-ACTGCCACTATCAAATTCTCGTGTGAAATCACCAGAAAAC-3’

To create a doxycycline-inducible lentivirus vector, the CA-IRF6 gene was PCR amplified from the modified pLenti6.3 IRF6 vector using the following primers:

FW: 5’-ACCGCTAGCCACCATGGCTTCTAGCTATCCTTATGAC-3’

RV: 5’- ACCACGCGTTCAGGATCCACTAGGGACTTTA-3’

The PCR product was double digested with restriction enzymes NheI and MluI and inserted into the pCW vector at the NheI and MluI restriction sites to obtain the pCW-CA-IRF6 vector.

To create the constitutively inactive doxycycline-inducible IRF6, the pCW-caIRF6 plasmid was amplified with the following primers:

FW: 5’-TTTCACACGAGCCTTTGATAGTGGCAGTGTCCGCCTGCCAGATCGCAACCCCAGACATCAA-3’

RV: 5’-TCACCAGAAAACATCTCGTAGATCCGAGCC-3’

The resulting product was subjected to the KLD reaction (NEB) and the ligated plasmid transformed into NEB Stable cells. Correct colonies were confirmed by whole plasmid sequencing at Plasmidsaurus.

To generate a doxycycline-inducible BRLF1 plasmid, a PCR fragment was generated from a plasmid containing AG876 BRLF1 using the following primers:

FW:5’-GCCGAATTCATGAGGCCTAAAAAGGATGGCTTGG-3’

RV:5’-GAAACGCGTCTAAAATAAGCTGGTGTCAAAAATAGACAGCC-3’,

The PCR product digested with EcoRI-HF and BamHI-HF (NEB) and ligated into pCW digested with the same enzymes.

To generate a doxycycline-inducible BZLF1 vector, a plasmid containing the cDNA for B95.8 BZLF1 was PCR amplified with the following primers:

FW:5’-GGGCGAATTCCGGTGAAGATGATGG-3’

RV:5’-GAAACGCGTTTAGAAATTTAAGAGATCCTCGTGTAAAACATCTGG-3’

The PCR product was digested with EcoRI-HF and MluI-HF, and ligated into pCW digested with the same enzymes.

All amplifications were performed with the Q5 polymerase (NEB) and all transformations were into NEB Stable E. coli.

All plasmids were confirmed by whole plasmid sequencing at Plasmidsaurus (Arcadia, CA) using Oxford Nanopore Technology with custom analysis and annotation and verified. All the primers were ordered from IDTDNA (Coralville IA).

### Lentivirus production, concentration, and transduction

Lentivirus vectors were packaged in LentiX HEK293 cells. To package, 4μg of lentivirus vector, 3μg of pSPAX2 (psPAX2 was a gift from Didier Trono (Addgene plasmid # 12260; http://n2t.net/addgene:12260; RRID:Addgene_12260), and 1μg of pMD2.G (pMD2.G was a gift from Didier Trono (Addgene plasmid # 12259; http://n2t.net/addgene:12259; RRID:Addgene_12259) vectors were transfected into a 60% confluent 10 cm cell culture dish of LentiX cells using 24μL of Lipofectamine 2000 (Thermo Fisher Ref#: 11668019) in 500μL of optimem. Twenty-four hours later, the transfected cells’ media was changed to the intended target cells’ media. This media was collected the next day, filtered with a 0.45μM syringe filter and combined with the 4X PEG 8000 lentivirus concentrator solution described by MD Anderson here: (https://BLIMP.mdanderson.org/documents/corefacilities/Functional%20Genomics%20Core/Homemade%204fold%20lentivirus%20concentrator.pdf). Once concentrated, the lentivirus was pelleted and resuspended per the above MD Anderson protocol in fresh, antibiotic free target cell media then placed on target cells overnight. Puromycin selection was started 48 hours later and continued until negative control target cells were killed. 500ng/mL doxycycline (for three days) was used to induce overexpression of CA-IRF6 or inactive IRF6 in these cells following selection.

### siRNA knock-down

siRNAs were transfected using RNAiMax (Thermo Fisher Ref# 13778150) at a final concentration of 20pM according to the manufacturer’s protocol. NOKs cells transfected with siRNA had their media changed 4 hours after transfection due to their sensitivity to lipofectamine toxicity. Depending on the efficiency of knock-down, cells were either lysed using SUMO lysis buffer 48 hours later or transfected with siRNA again at 48 hours and lysed at 72 hours after the initial siRNA transfection.

siRNAs used: IRF6 (Santa Cruz sc-105582), IRF6 (Dharmacon L-012227-00-0005), PKCδ (Santa Cruz sc-36253), PKCδ (Dharmacon L-003524-00-0005), RIPK4 (Santa Cruz sc-91500), RIPK4 (Dharmacon M-005308-03-0005), BLIMP1 (Santa Cruz sc-37714), BLIMP1 (Dharmacon L-009322-00-0005), KLF4 (Santa Cruz sc-35480), KLF4 (Dharmacon L-005089-00-0005), non-targeting control (Santa Cruz sc-44230) and non-targeting control (Dharmacon D-001810-10-05).

All siRNAs used were pools, with each target gene targeted by a pool from two different companies, Santa Cruz Biotechnology and Horizon Discovery/Dharmacon. In each instance, the siRNA pool labeled as “#1” was from Santa Cruz, while the siRNA pool labeled as “#2” was from Horizon/Dharmacon.

### Chemicals

Phorbol 12-myristate 13-acetate (TPA) (Sigma # P8139) was dissolved in DMSO and used at 20ng/mL. Rock inhibitor Y-27632 (R&D Systems #1254/10) was dissolved in RPMI 1640 and used at 4μM. Doxycycline hyclate (Sigma-Aldrich # D-5207) was dissolved in water, used at 500ng/mL, and given daily. For each chemical, where relevant, the control condition was equal volumes of solvent.

### Immunoblotting

Immunoblots were performed according to the methods previously described [44]. A summary of this protocol is as follows: cell lysates were created using SUMO lysis buffer (1:3 ratio of SUMO buffer I [5% SDS, 30% glycerol, 0.15M Tris-HCl (pH 6.8)] and SUMO buffer II [0.1% SDS, 0.5% deoxycholate, 0.5% NP-40, 50mM NaCl, 25mM Tris-HCl (pH 8.3]) with Roche cOmplete protease inhibitor added. Protein concentration of lysates was quantitated with the DC Bio-Rad protein assay. Proteins were separated on appropriate percentage (8–15%) SDS-PAGE gel and transferred to nitrocellulose membrane. Blocking of background on these membranes was performed using 5% milk dissolved in 1X PBS with 0.1% Tween 20 for one hour. After blocking, membranes were incubated at 4°C overnight in primary antibody. Following this incubation, blots were washed in wash buffer (1X PBS 0.1% Tween 20) three times for five minutes. Next, blots were incubated in secondary antibody suspended in 5% milk dissolved in wash buffer for one hour. Blots were next washed three times for 15 minutes in wash buffer then treated with ECL and imaged using film.

### Immunohistochemistry

NOKs cells plated on acid-washed coverslips were washed with PBS, then fixed for 30 minutes at room temperature with 10% formalin/PBS. The fixative was removed and the cells washed three times with PBS and stored at 4^o^C until staining. To stain, the PBS was removed and the cells blocked for 30 minutes in 10% Normal Horse Serum (NHS) (Vector Labs, Burlingame CA), 0.3% H_2_O_2_, 1% Triton X-100. The block was removed and the cells stained overnight at 4^o^C with either a 1:5000 dilution of BZLF1 antibody (Santa Cruz #sc-53904) or a 1:2000 dilution of IRF6 antibody (Biolegend #674502), in 10% NHS, PBS, 0.1% Tween-20. The following day, the antibody was removed and the cells washed 2x10 minutes PBS-T. The cells were then incubated for 30minutes with Vector ImmPRESS HRP horse anti-mouse (Vector Laboratories # MP-7402), washed 2x10 minutes PBS and developed 1–3 minutes with SignalStain DAB substrate Kit (Cell Signaling #8059), the coverslips dehydrated through graded alcohols, cleared in xylene and mounted on slides.

### Immunoblot antibodies

Primary antibodies used: IRF6 (Biolegend #674502), PKCδ (Cell Signaling #2508), phospho-PKCδ (Cell Signaling #2055), RIPK4 (Abnova #H00054101-m01), BLIMP1 (Cell Signaling #9115), KLF4 (Cell Signaling #12173), Involucrin (Sigma #9018), Keratin 10 (Biolegend #905404), BZLF1 (Santa Cruz #sc-53904), a BRLF1 rabbit polyclonal antibody directed against the BRLF1 peptide sequence EDPDEETSSQAVKALREMAD (BRLF1 residues 506–524) generated by Pierce Biotechnology, BMRF1 (Milipore Sigma #MAB8186), a p18 VCA Rabbit polyclonal antibody directed against the p18 VCA peptide sequence: GQPQDTAPRGARKKQ (p18 VCA residues 162–176), generated by Thermo Scientific, ELF3 (Sigma #HPA003479), TGM1 (Novus #34062), ΔNp63α (Cell signaling #13109), SPRR1A (abclonal technology # A17535), CHOP (Cell Signaling # 2895), ZNF750 (Sigma # HPA023012), GRHL3 (Novus # NBP1–80356), Tubulin (Sigma #T5168), β-Actin (Sigma #A5441).

Secondary antibodies used: HRP-conjugated goat anti-mouse (Thermo Scientific #31430 and HRP-conjugated goat anti-rabbit (Fisher Scientific).

## Supporting information

S1 Fig IRF6 expression is required for spontaneous, and TPA-induced, lytic EBV infection and TPA-induced differentiation in NOKs. (**A)** Uninfected NOKs (NOKs (-)) or NOKs infected with AG876 type 2 strain EBV (NOKs EBV(+)) were plated in the absence of growth factors in KSFM, at a sub-confluent density, treated with a control siRNA or an IRF6-directed siRNA for two days, and then treated with or without TPA for 24 hours before harvesting protein extracts for immunoblot analysis. Expression levels of IRF6, the EBV lytic proteins BZLF1, BRLF1, BMRF1, and LMP1 and epithelial differentiation markers BLIMP1 and ELF3, are shown. Tubulin served as a loading control. Note that different control siRNA and IRF6 siRNA were used in this experiment compared to the experiments shown in Fig 1.
(TIF)

S2 Fig Knock-down of IRF6, with and without TPA treatment, does not cause any significant toxicity in EBV-infected NOKs. NOKs infected with AG876 type 2 strain EBV (NOKs EBV(+)) were plated in the absence of growth factors in KSFM, at a sub-confluent density, treated with a control siRNA or an IRF6-directed siRNA for two days, and then treated with or without TPA for 24 hours and brightfield images were taken to assess cell health.
(TIF)

S3 Fig IRF6 expression is required for TPA-induced, lytic EBV reactivation in EBV-infected nasopharyngeal carcinoma NPC43 and gastric carcinoma SNU719 cell lines. (**A)** NPC43 cells were treated with a control siRNA or an IRF6-directed siRNA for 24 hours, then treated with or without TPA for 24 hours before harvesting protein extracts for immunoblot analysis. Expression levels of IRF6 and the EBV lytic proteins BZLF1 and BMRF1 are shown. Tubulin served as a loading control. **(B)** SNU719 cells were treated with a control siRNA or an IRF6-directed siRNA for 24 hours, then treated with or without TPA for 24 hours before harvesting protein extracts for immunoblot analysis. Expression levels of IRF6 and the EBV lytic proteins BZLF1 and BMRF1 are shown. Tubulin served as a loading control. Note that different control siRNA and IRF6 siRNA were used in this experiment compared to the experiments shown in Fig 2.
(TIF)

S4 Fig PKCδ expression is required for spontaneous, and TPA-induced, lytic EBV reactivation and TPA-induced differentiation in NOKs. Uninfected NOKs (NOKs (-)) or NOKs infected with type 2 strain AG876 EBV (NOKs EBV(+)) were plated in the absence of growth factors in KSFM, at a sub-confluent density, treated with a control siRNA or a PKCδ-directed siRNA for two days, and then treated with or without TPA for 24 hours before harvesting protein extracts for immunoblot analysis. Expression levels of PKCδ, the EBV lytic proteins BRLF1, BZLF1, BMRF1, and LMP1, and the differentiation markers IRF6, BLIMP1, and ELF3 are shown. Tubulin served as a loading control. Note that different control siRNA and IRF6 siRNA were used in this experiment compared to the experiments shown in Fig 3.
(TIF)

S5 Fig RIPK4 expression is required for TPA-induced lytic EBV reactivation and differentiation in NOKs. NOKs infected with Akata type 1 strain EBV (NOKs EBV(+)) were plated in the absence of growth factors in KSFM, at a sub-confluent density, treated with a control siRNA or a RIPK4-directed siRNA for two days, and then treated with or without TPA for 24 hours before harvesting protein extracts for immunoblot analysis. Expression levels of IRF6, and the epithelial differentiation marker, BLIMP1, are shown, as well as the lytic EBV proteins BRLF1, BZLF1 and BMRF1. Tubulin served as a loading control. The size of phosphorylated RIPK4 is indicated by “P”. Note that different control siRNA and IRF6 siRNA were used in this experiment compared to the experiments shown in Fig 4.
(TIF)

S6 Fig Constitutively active IRF6 does not induce CHOP expression in EBV-infected NOKs. Akata type 1 strain EBV-infected NOKs were stably infected with a control vector or a lentivirus expressing a doxycycline inducible phospho-mimetic IRF6 mutant (CA-IRF6, in which serine residues 413 and 424 were switched to glutamic acid) and then treated with 500ng/mL doxycycline for three days and immunoblot analyses were performed to examine expression of IRF6 and CHOP as shown. NOKs EBV(+) cells were also treated with tunicamycin to act as a positive control for CHOP expression. Actin served as a loading control.
(TIF)

S7 Fig Constitutively active IRF6 induces lytic EBV reactivation and epithelial cell differentiation in EBV-infected NOKs. Akata type 1 strain EBV-infected NOKs were stably infected with a control vector or a lentivirus expressing a doxycycline inducible phospho-mimetic IRF6 mutant (CA-IRF6, in which serine residues 413 and 424 were switched to glutamic acid), selected with puromycin for five days, and then treated with 500ng/mL doxycycline for 72 hours and examined by immunoblot analyses to examine expression of IRF6, lytic EBV proteins BZLF1, BRLF1, and LMP1, and the differentiation markers Involucrin, BLIMP1, KLF4, and ΔNp63α as shown. Tubulin served as a loading control.
(TIF)

S8 Fig Constitutively active IRF6 does not cause significant toxicity in EBV-infected NOKs. Akata type 1 strain EBV-infected NOKs were infected with a control vector or a lentivirus expressing a doxycycline inducible phospho-mimetic IRF6 mutant (CA-IRF6, in which serine residues 413 and 424 were switched to glutamic acid), then treated with 500ng/mL doxycycline for 72 hours and brightfield images were taken to examine cell health.
(TIF)

S9 Fig Doxycycline treatment induces expression of constitutively active IRF6 in a portion of EBV infected NOKs cells, and this induces the expression of the EBV lytic protein BZLF1. Akata type 1 EBV strain-infected NOKs were infected with a control vector or a lentivirus expressing a doxycycline inducible phospho-mimetic IRF6 mutant (CA-IRF6, in which serine residues 413 and 424 were switched to glutamic acid), plated on coverslips, then treated with 500ng/mL doxycycline for 72 hours. Cells were fixed using 3.7% formaldehyde, and stained using immunohistochemistry to detect IRF6 or BZLF1 expression and brightfield images were taken. Two separate fields stained with the anti-BZLF1 or anti-IRF6 antibodies from the same experiment are shown.
(TIF)

S10 Fig Constitutively active IRF6 induces lytic EBV reactivation in EBV-infected SNU719 gastric carcinoma cells. EBV-infected SNU719 cells were infected with a control vector or a lentivirus expressing a doxycycline inducible phospho-mimetic IRF6 mutant (CA-IRF6, in which serine residues 413 and 424 were switched to glutamic acid) then treated with 500ng/mL doxycycline for 72 hours and examined by immunoblot analyses to examine expression of IRF6, the lytic EBV protein BZLF1, and the epithelial cell differentiation markers BLIMP1 and KLF4 as shown. Tubulin served as a loading control.
(TIF)

S11 Fig BLIMP1 and KLF4 expression are required for CA-IRF6-induced lytic EBV reactivation in NOKs. Akata type 1 EBV strain-infected NOKs were infected with a control vector or a lentivirus expressing a doxycycline inducible phospho-mimetic IRF6 mutant (CA-IRF6) and then transfected with control siRNA or siRNAs directed to KLF4 or BLIMP1 as indicated. 24h following transfection, cells were treated with 500ng/ml doxycycline for 72 hours and immunoblot analyses were performed to examine expression of CA-IRF6, BLIMP1, KLF4, BZLF1, and BRLF1 as shown. Actin serves as a loading control. Note that different control siRNA and IRF6 siRNA were used in this experiment compared to the experiments shown in Fig 8.
(TIF)

S12 Fig BZLF1 and BRLF1 expression bypasses inhibitory effect of IRF6 knock-down in TPA treated EBV-infected NOKs cells. Akata type 1 EBV strain infected NOKs were stably infected with a control vector or a lentiviruses expressing doxycycline inducible BRLF1 alone or the combination of BRLF1 and BZLF1 EBV IE proteins, and then transfected with control siRNA or siRNAs directed to IRF6 as indicated. 24h following transfection, cells were treated with 500ng/ml doxycycline for 24 hours with and without TPA and immunoblot analyses were performed to examine expression of IRF6, BZLF1, BRLF1 and BMRF1 as shown. Tubulin serves as a loading control. The sizes of the BZLF1 protein (B95.8 strain) produced following BZLF1 lentivirus vector infection versus the endogenous (Akata strain) BZLF1 protein induced by TPA treatment are indicated.
(TIF)
