# Correction: IRF6 controls Epstein-Barr virus (EBV) lytic reactivation and differentiation in EBV-infected epithelial cells

**DOI:** 10.1371/journal.ppat.1013564

**Published:** 2025-10-09

**Authors:** The PLOS Pathogens Staff

Figs 1–9 were uploaded incorrectly. Please see the correct Figs 1–9 here.

**Fig 1:**
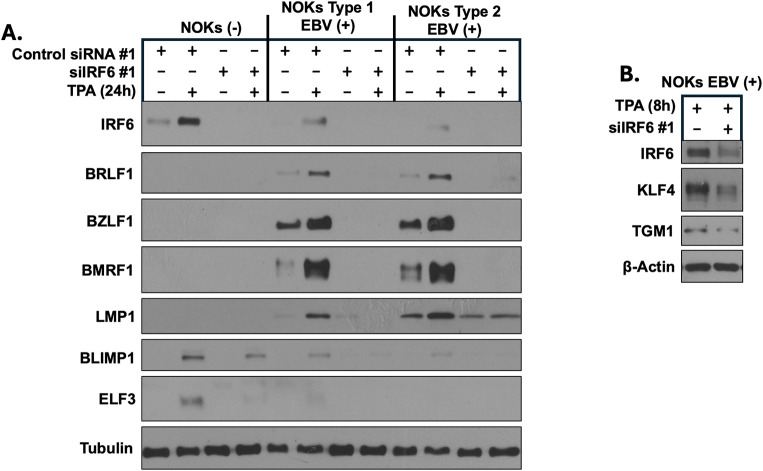
IRF6 expression is required for spontaneous, and TPA-induced, lytic EBV infection and TPA-induced differentiation in NOKs. **(A)** Uninfected NOKs (NOKs (-)) or NOKs infected with AG876 type 2 strain EBV or Akata type 1 strain EBV were plated in the absence of growth factors in KSFM, at a sub-confluent density, treated with a control siRNA or an IRF6-directed siRNA for two days, and then treated with or without TPA for 24 hours before harvesting protein extracts for immunoblot analysis. Expression levels of IRF6, the EBV IE lytic proteins BZLF1 and BRLF1, the early lytic proteins BMRF1 and LMP1, and epithelial differentiation markers BLIMP1 and ELF3, are shown. Tubulin served as a loading control. **(B)** AG876 EBV-infected NOKs were plated in the absence of growth factors in KSFM, at a sub-confluent density, treated with a control siRNA or an IRF6-directed siRNA for two days, and then treated with or without TPA for 8 hours before harvesting protein extracts for immunoblot analysis. Expression levels of IRF6 and the differentiation markers, KLF4 and TGM1, are shown. Actin served as a loading control.

**Fig 2:**
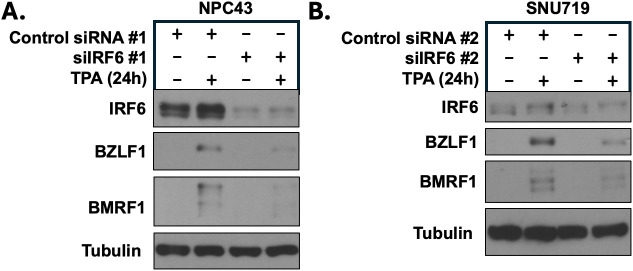
IRF6 expression is required for TPA-induced lytic EBV infection in EBV-infected nasopharyngeal carcinoma NPC43 and gastric carcinoma SNU719 cells. **(A)** NPC43 cells were treated with a control siRNA or an IRF6-directed siRNA for 24 hours, then treated with or without TPA for 24 hours before harvesting protein extracts for immunoblot analysis. Expression levels of IRF6 and the EBV lytic proteins BZLF1 and BMRF1 are shown. Tubulin served as a loading control. **(B)** SNU719 cells were treated with a control siRNA or an IRF6-directed siRNA for 24 hours, then treated with or without TPA for 24 hours before harvesting protein extracts for immunoblot analysis. Expression levels of IRF6 and the EBV lytic proteins BZLF1 and BMRF1 are shown. Tubulin served as a loading control.

**Fig 3:**
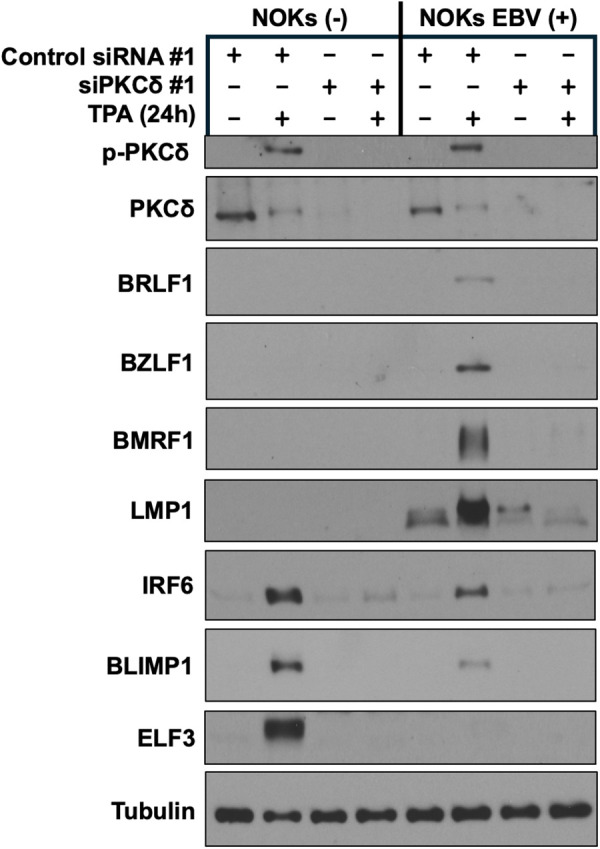
PKCδ expression is required for TPA-induced lytic EBV infection and TPA-induced differentiation in NOKs. Uninfected NOKs (NOKs (-)) or NOKs infected with AG876 type 2 strain EBV (NOKs EBV(+)) were plated in the absence of growth factors in KSFM, at a sub-confluent density, treated with a control siRNA or a PKCδ-directed siRNA for two days, and then treated with or without TPA for 24 hours before harvesting protein extracts for immunoblot analysis. Expression levels of PKCδ, the EBV lytic proteins BRLF1, BZLF1, BMRF1, and LMP1, and the differentiation markers IRF6, BLIMP1, and ELF3 are shown. Tubulin served as a loading control.

**Fig 4:**
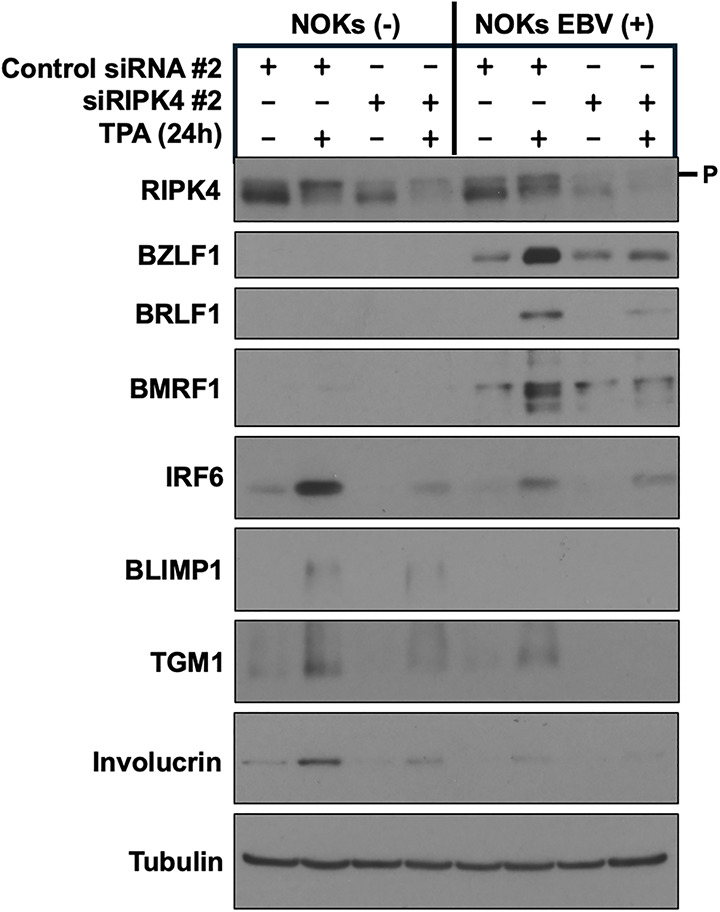
RIPK4 expression is required for TPA-induced lytic EBV infection and TPA-induced differentiation in NOKs. Uninfected NOKs (NOKs (-)) or NOKs infected with AG876 type 2 strain EBV (NOKs EBV(+)) were plated in the absence of growth factors in KSFM, at a sub-confluent density, treated with a control siRNA or a RIPK4-directed siRNA for two days, and then treated with or without TPA for 24 hours before harvesting protein extracts for immunoblot analysis. Expression levels of RIPK4, the EBV lytic proteins BRLF1, BZLF1, and BMRF1, and the differentiation markers IRF6, BLIMP1, TGM1, and involucrin are shown. Tubulin served as a loading control. The size of phosphorylated RIPK4 is indicated by “P”.

**Fig 5:**
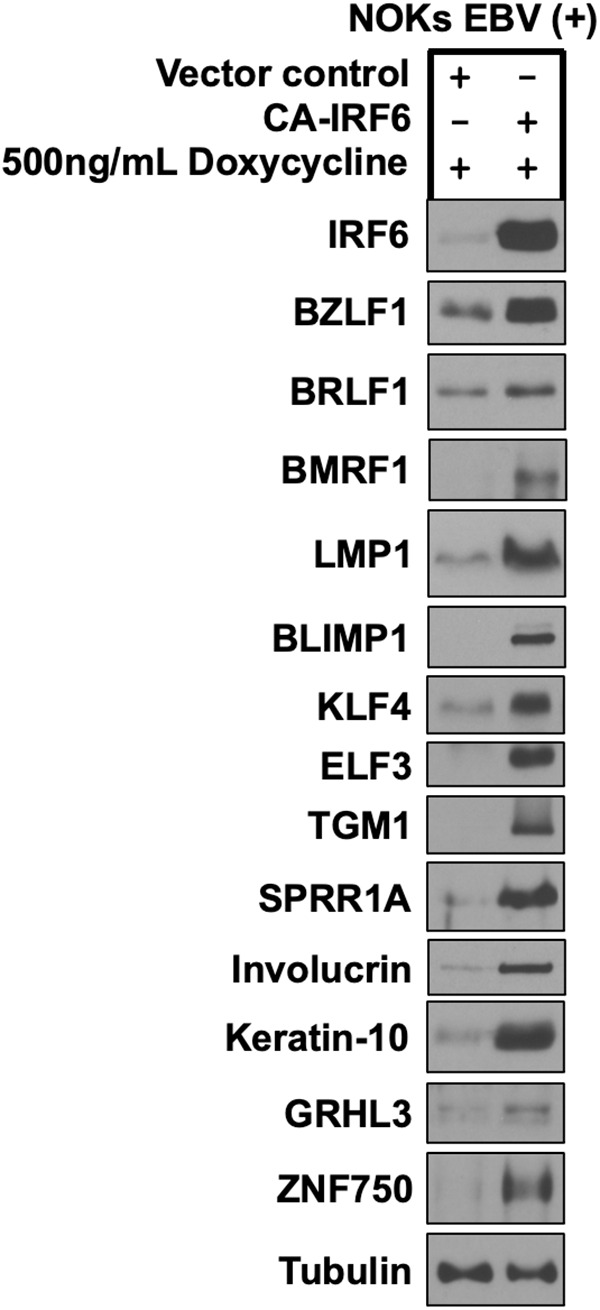
Constitutively active IRF6 induces lytic EBV reactivation and epithelial cell differentiation in EBV-infected NOKs. AG876 type 2 strain EBV-infected NOKs were stably infected with a control vector or a lentivirus expressing a doxycycline inducible phospho-mimetic IRF6 mutant (CA-IRF6, in which serine residues 413 and 424 were switched to glutamic acid), selected with puromycin for five days, and then treated with 500ng/mL doxycycline for 72 hours and examined by immunoblot analyses to examine expression of IRF6, lytic EBV proteins BZLF1, BRLF1, BMRF1, and LMP1, and the differentiation markers BLIMP1, KLF4, ELF3, TGM1, SPRR1A, Involucrin, Keratin 10, GRHL3, and ZNF750 as shown. Tubulin served as a loading control.

**Fig 6:**
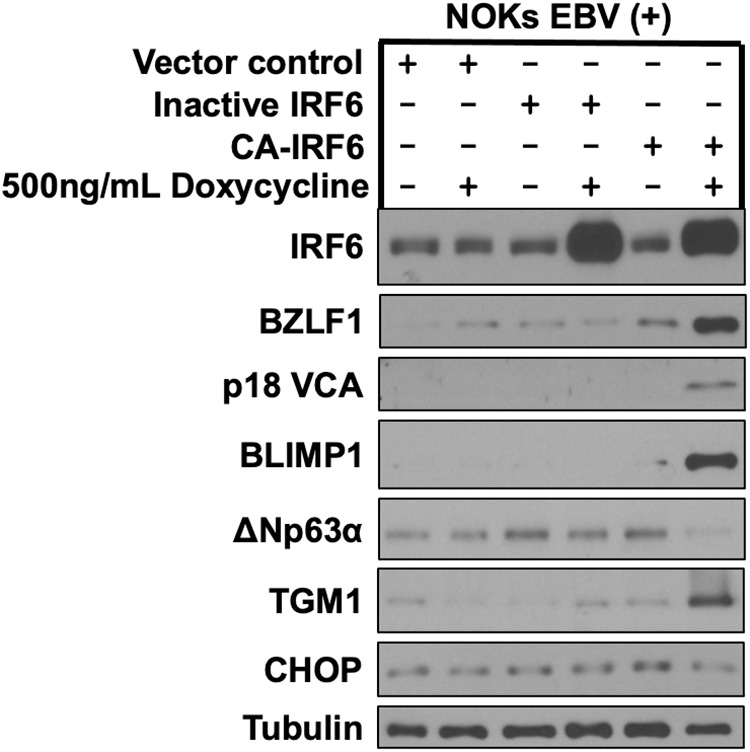
An inactive IRF6 mutant does not induce lytic EBV reactivation and epithelial cell differentiation in EBV-infected NOKs. Akata type 1 strain EBV-infected NOKs were stably infected with a control vector or a lentivirus expressing a doxycycline inducible phospho-mimetic IRF6 mutant (CA-IRF6, in which serine residues 413 and 424 were switched to glutamic acid) or an inactive IRF6 mutant (Inactive IRF6, in which serine residues 413 and 424 were switched to alanine), then treated with 500ng/mL doxycycline for 72 hours and examined by immunoblot analyses to examine expression of IRF6, the IE lytic EBV protein BZLF1 and the late lytic EBV protein p18 VCA, the differentiation markers BLIMP1, ΔNp63α, and TGM1, and the integrated stress response protein CHOP as shown. Tubulin served as a loading control.

**Fig 7:**
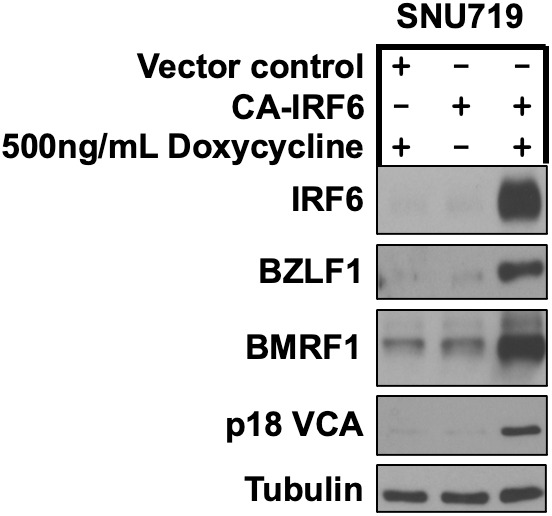
Constitutively active IRF6 induces lytic EBV reactivation in EBV-infected SNU719 gastric carcinoma cells. EBV-infected SNU719 cells were stably infected with a control vector or a lentivirus expressing a doxycycline inducible phospho-mimetic IRF6 mutant (CA-IRF6, in which serine residues 413 and 424 were switched to glutamic acid) then treated with 500ng/mL doxycycline for 72 hours and examined by immunoblot analyses to examine expression of IRF6 and IE lytic EBV protein BZLF1, the early lytic EBV protein BMRF1, and the late lytic EBV protein p18 VCA as shown. Tubulin served as a loading control.

**Fig 8:**
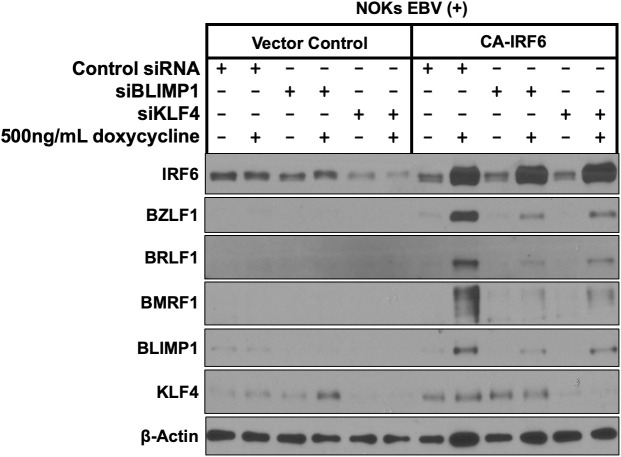
BLIMP1 and KLF4 expression are required for CA-IRF6-induced lytic EBV reactivation in NOKs. NOKs infected with type 1 strain Akata EBV (NOKs EBV(+)) were stably infected with a control vector or a lentivirus expressing a doxycycline inducible phospho-mimetic IRF6 mutant (CA-IRF6) and then transfected with control siRNA or siRNAs directed to KLF4 or BLIMP1 as indicated. 24h following transfection, cells were treated with 500ng/ml doxycycline for 72 hours and immunoblot analyses were performed to examine expression of CA-IRF6, BLIMP1, KLF4, and lytic EBV proteins BZLF1, BRLF1 and BMRF1 as shown. Actin serves as a loading control.

**Fig 9:**
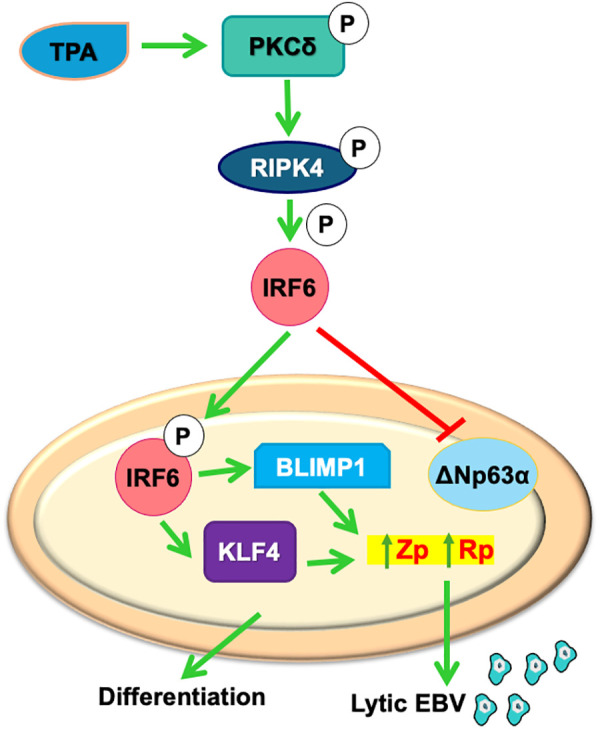
Model of TPA-induced activation of a PKCδ/ RIPK4/IRF6 signaling pathway inducing lytic EBV reactivation and epithelial cell differentiation in EBV-infected NOKs.

The publisher apologizes for the errors.
