# Supplementary figures and images for: IRF6 controls Epstein-Barr virus (EBV) lytic reactivation and differentiation in EBV-infected epithelial cells
Source: PLoS Pathog. 2025 Jun 26;21(6):e1013236. doi: 10.1371/journal.ppat.1013236 (PMC12200665; doi:10.1371/journal.ppat.1013236)

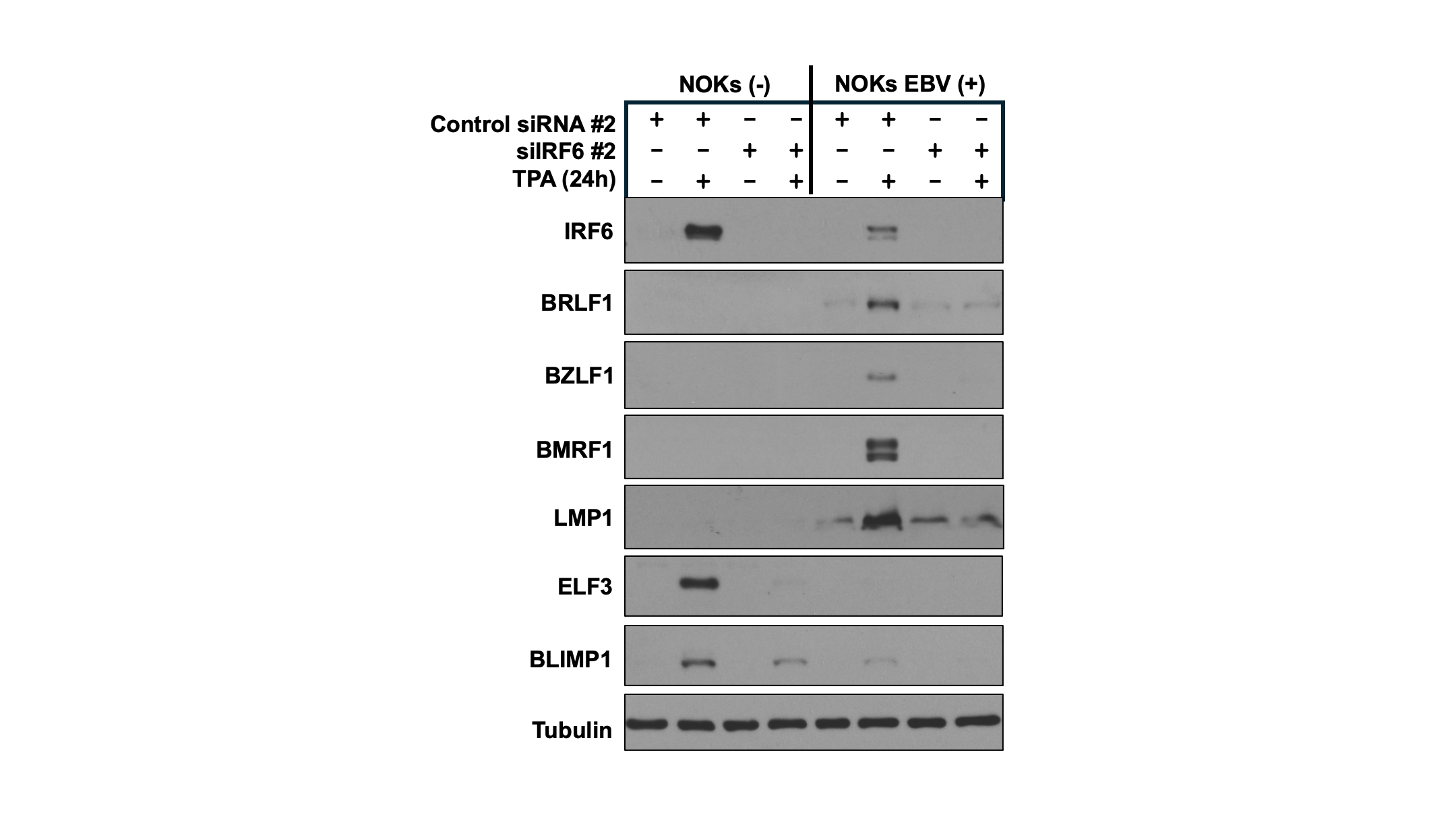

Supplement: S1 Fig — (A) Uninfected NOKs (NOKs (-)) or NOKs infected with AG876 type 2 strain EBV (NOKs EBV(+)) were plated in the absence of growth factors in KSFM, at a sub-confluent density, treated with a control siRNA or an IRF6-directed siRNA for two days, and then treated with or without TPA for 24 hours before harvesting protein extracts for immunoblot analysis. Expression levels of IRF6, the EBV lytic proteins BZLF1, BRLF1, BMRF1, and LMP1 and epithelial differentiation markers BLIMP1 and ELF3, are shown. Tubulin served as a loading control. Note that different control siRNA and IRF6 siRNA were used in this experiment compared to the experiments shown in Fig 1. (TIF) [file ppat.1013236.s001.tif]

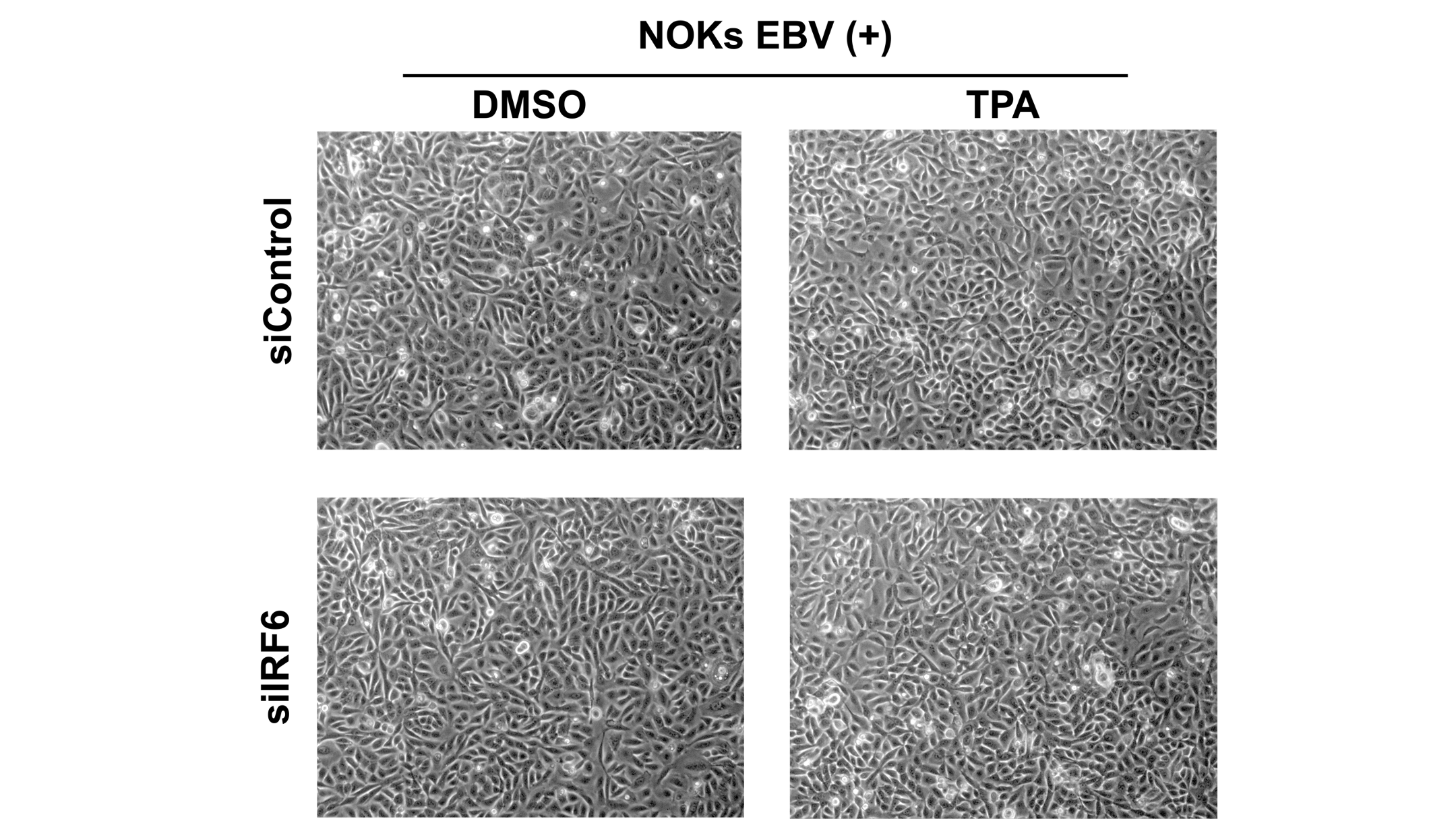

Supplement: S2 Fig — NOKs infected with AG876 type 2 strain EBV (NOKs EBV(+)) were plated in the absence of growth factors in KSFM, at a sub-confluent density, treated with a control siRNA or an IRF6-directed siRNA for two days, and then treated with or without TPA for 24 hours and brightfield images were taken to assess cell health. (TIF) [file ppat.1013236.s002.tif]

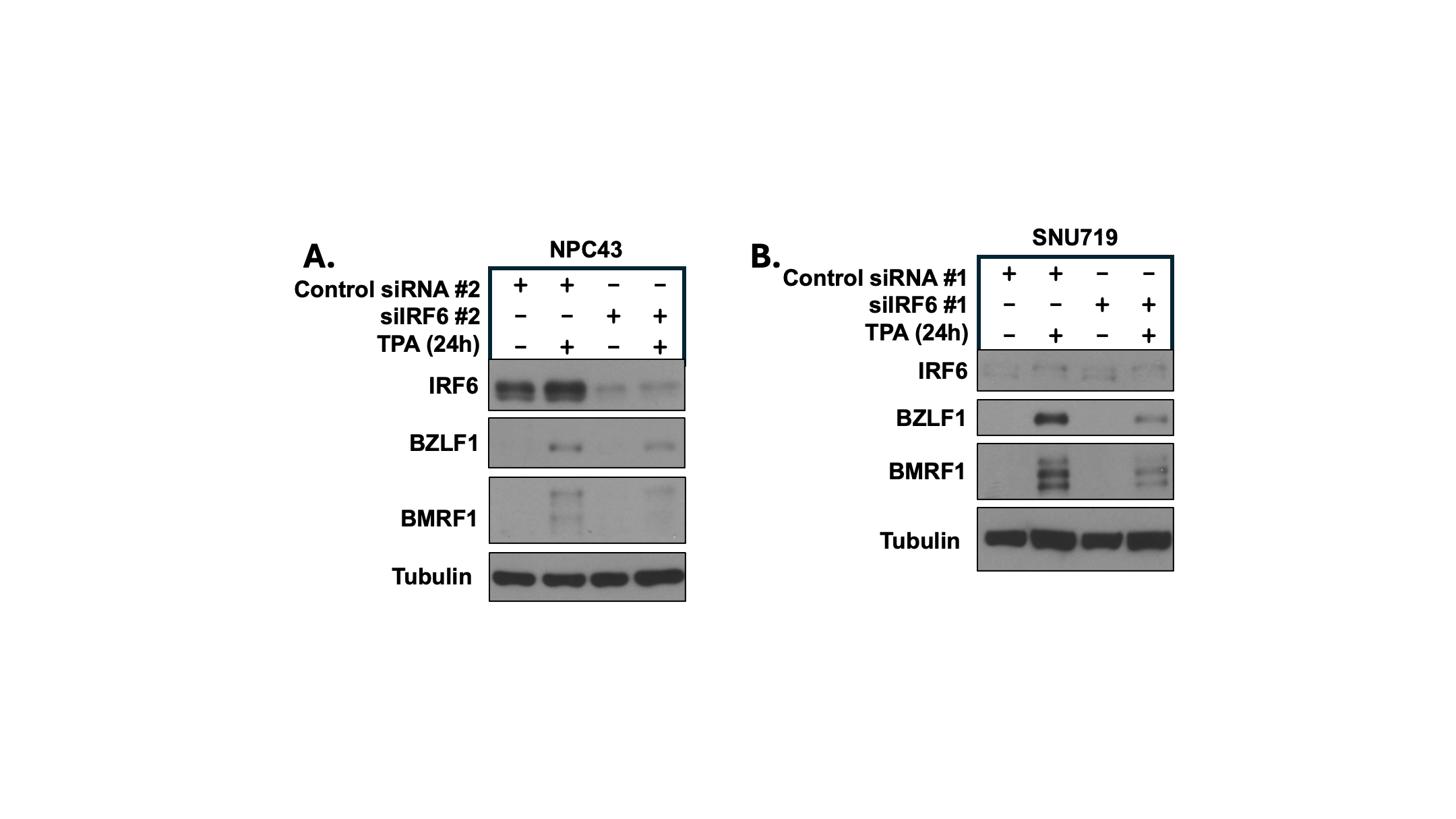

Supplement: S3 Fig — (A) NPC43 cells were treated with a control siRNA or an IRF6-directed siRNA for 24 hours, then treated with or without TPA for 24 hours before harvesting protein extracts for immunoblot analysis. Expression levels of IRF6 and the EBV lytic proteins BZLF1 and BMRF1 are shown. Tubulin served as a loading control. (B) SNU719 cells were treated with a control siRNA or an IRF6-directed siRNA for 24 hours, then treated with or without TPA for 24 hours before harvesting protein extracts for immunoblot analysis. Expression levels of IRF6 and the EBV lytic proteins BZLF1 and BMRF1 are shown. Tubulin served as a loading control. Note that different control siRNA and IRF6 siRNA were used in this experiment compared to the experiments shown in Fig 2. (TIF) [file ppat.1013236.s003.tif]

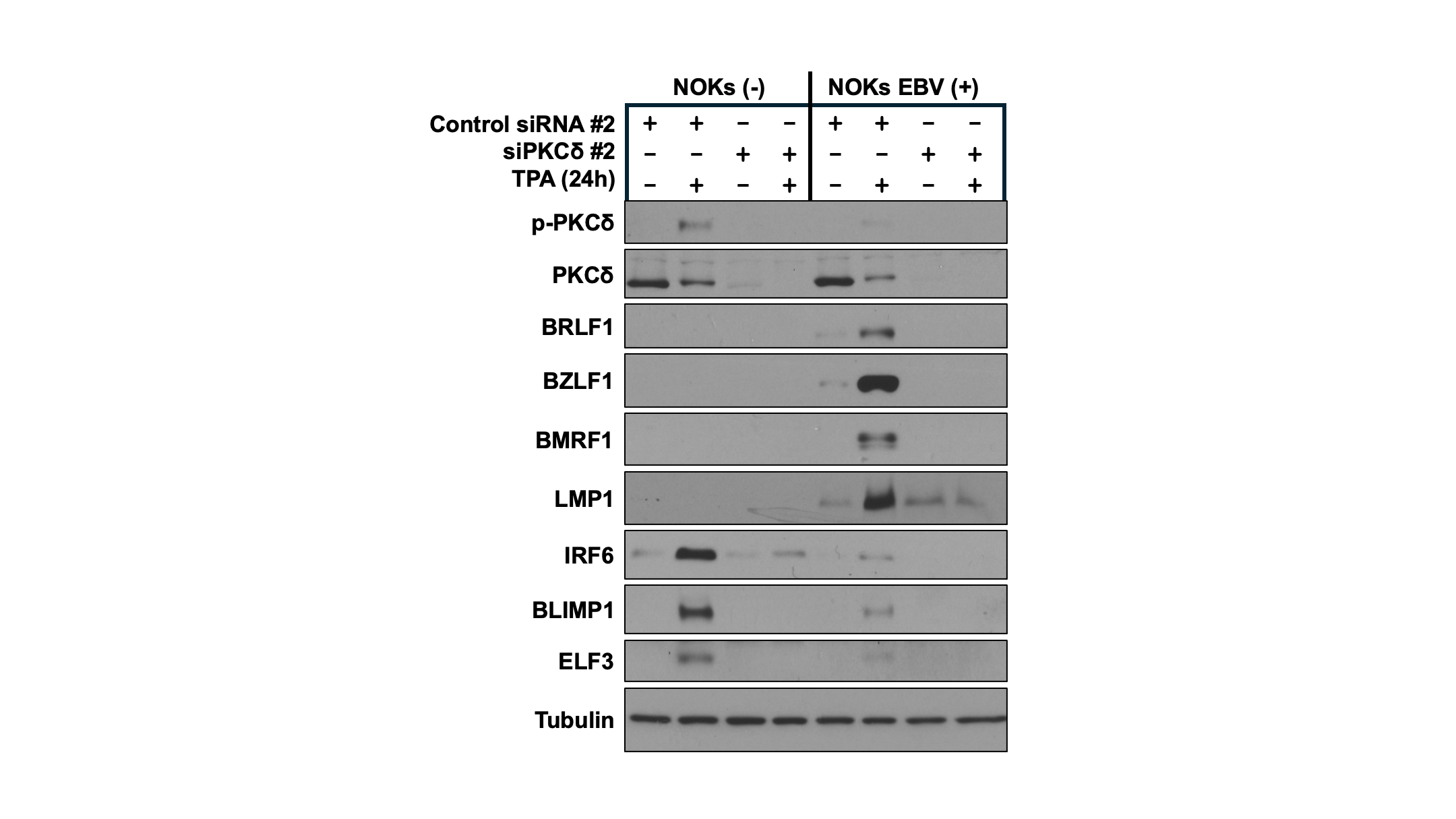

Supplement: S4 Fig — Uninfected NOKs (NOKs (-)) or NOKs infected with type 2 strain AG876 EBV (NOKs EBV(+)) were plated in the absence of growth factors in KSFM, at a sub-confluent density, treated with a control siRNA or a PKCδ-directed siRNA for two days, and then treated with or without TPA for 24 hours before harvesting protein extracts for immunoblot analysis. Expression levels of PKCδ, the EBV lytic proteins BRLF1, BZLF1, BMRF1, and LMP1, and the differentiation markers IRF6, BLIMP1, and ELF3 are shown. Tubulin served as a loading control. Note that different control siRNA and IRF6 siRNA were used in this experiment compared to the experiments shown in Fig 3. (TIF) [file ppat.1013236.s004.tif]

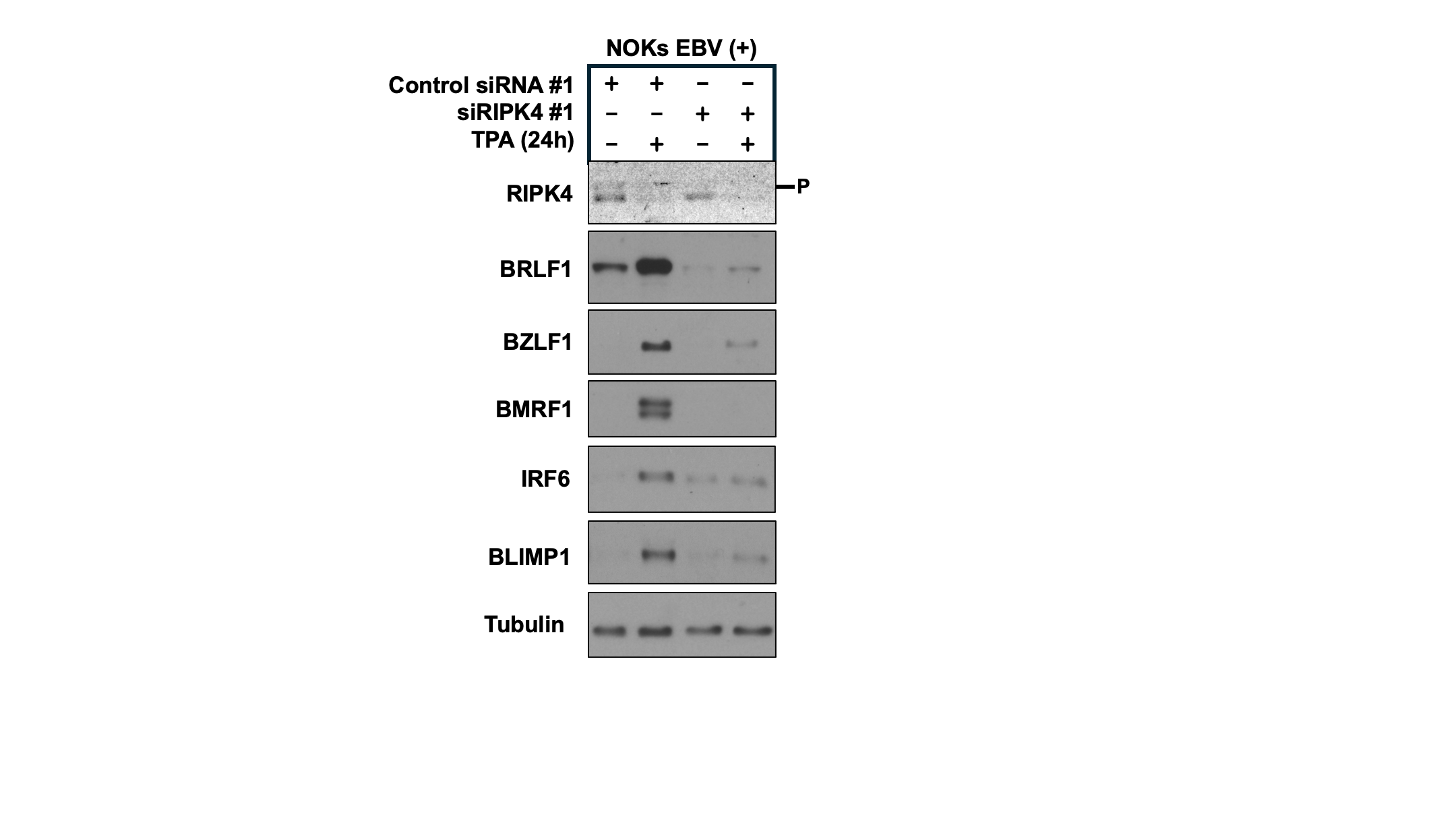

Supplement: S5 Fig — NOKs infected with Akata type 1 strain EBV (NOKs EBV(+)) were plated in the absence of growth factors in KSFM, at a sub-confluent density, treated with a control siRNA or a RIPK4-directed siRNA for two days, and then treated with or without TPA for 24 hours before harvesting protein extracts for immunoblot analysis. Expression levels of IRF6, and the epithelial differentiation marker, BLIMP1, are shown, as well as the lytic EBV proteins BRLF1, BZLF1 and BMRF1. Tubulin served as a loading control. The size of phosphorylated RIPK4 is indicated by “P”. Note that different control siRNA and IRF6 siRNA were used in this experiment compared to the experiments shown in Fig 4. (TIF) [file ppat.1013236.s005.tif]

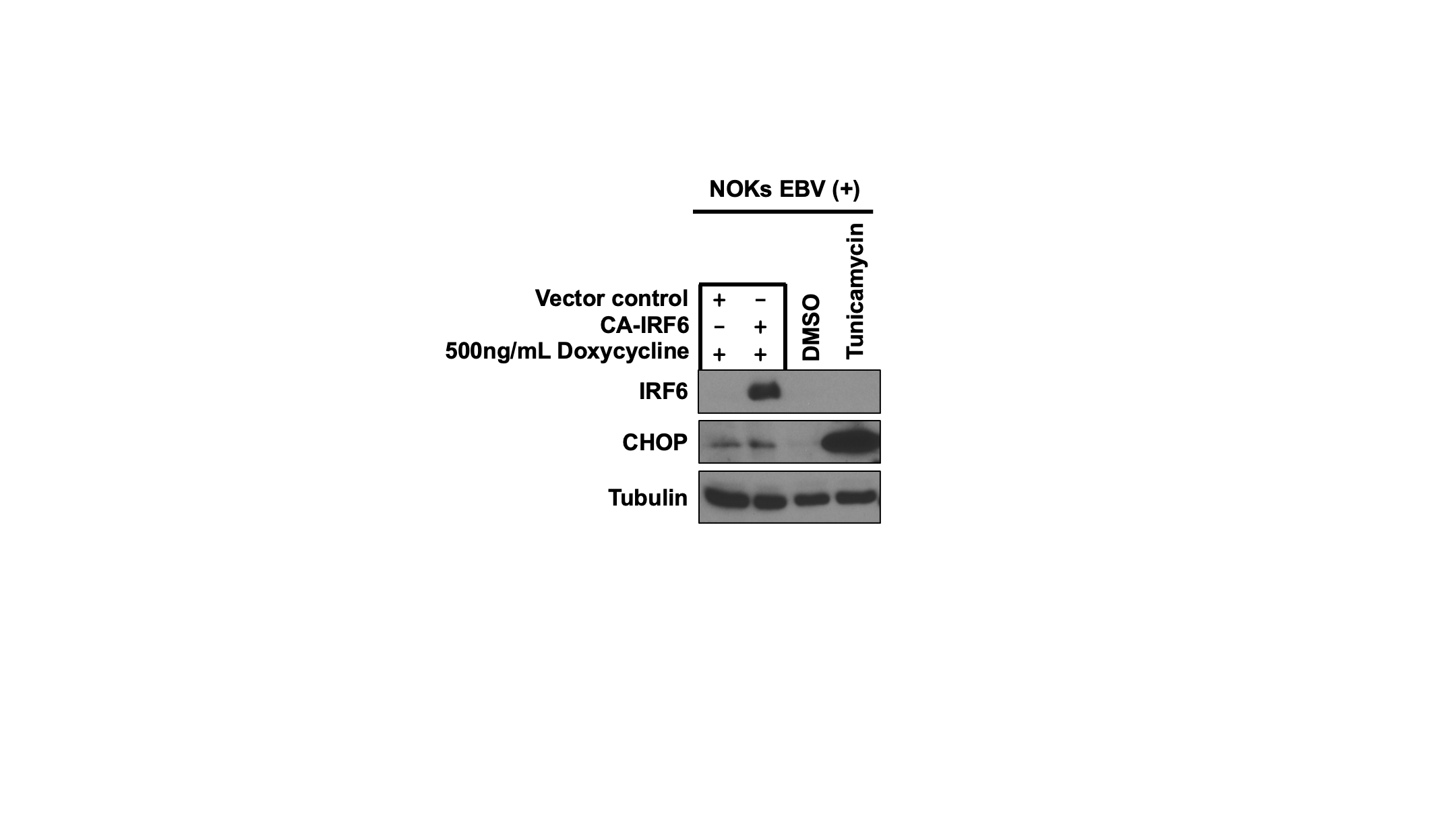

Supplement: S6 Fig — Akata type 1 strain EBV-infected NOKs were stably infected with a control vector or a lentivirus expressing a doxycycline inducible phospho-mimetic IRF6 mutant (CA-IRF6, in which serine residues 413 and 424 were switched to glutamic acid) and then treated with 500ng/mL doxycycline for three days and immunoblot analyses were performed to examine expression of IRF6 and CHOP as shown. NOKs EBV(+) cells were also treated with tunicamycin to act as a positive control for CHOP expression. Actin served as a loading control. (TIF) [file ppat.1013236.s006.tif]

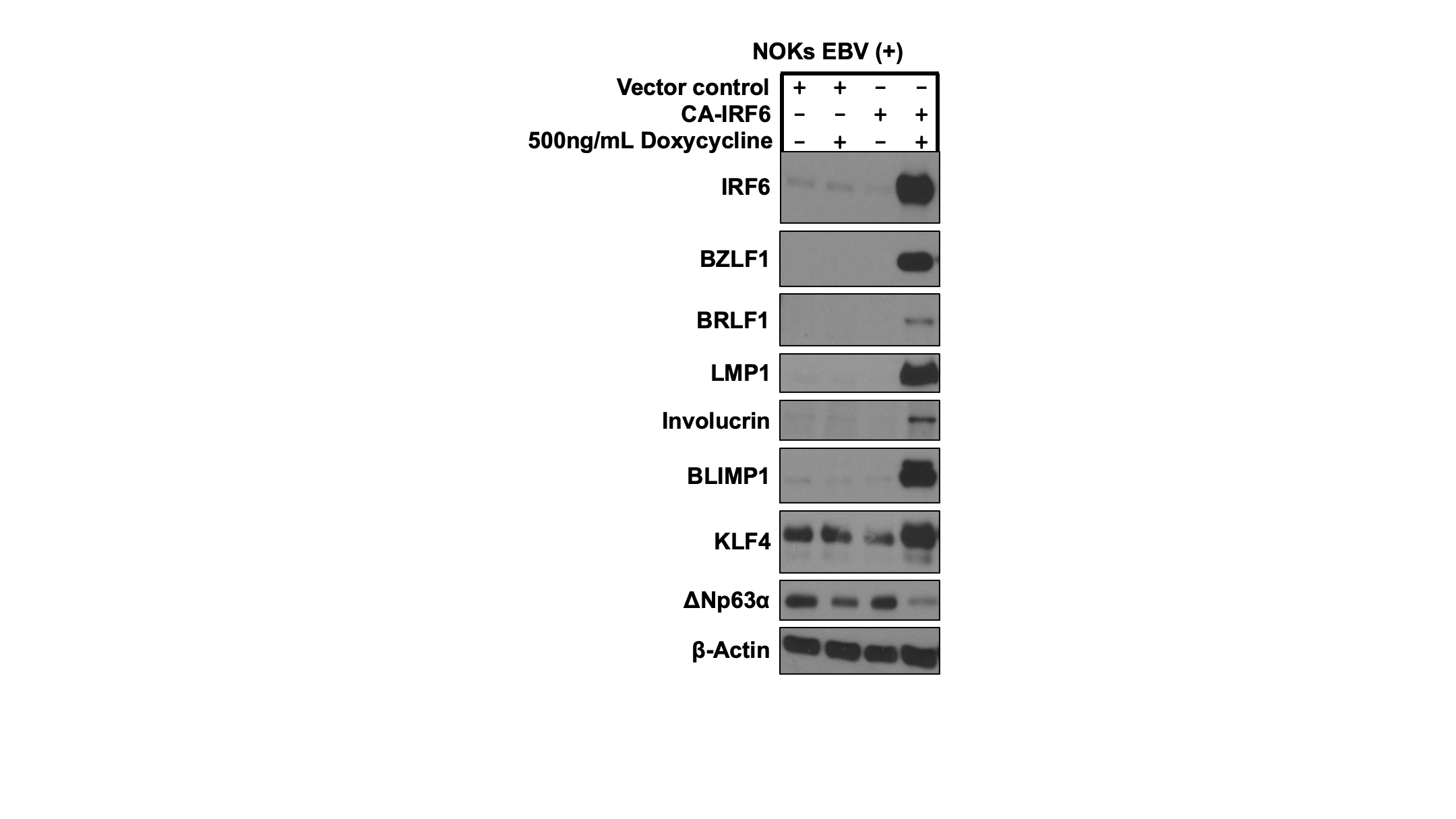

Supplement: S7 Fig — Akata type 1 strain EBV-infected NOKs were stably infected with a control vector or a lentivirus expressing a doxycycline inducible phospho-mimetic IRF6 mutant (CA-IRF6, in which serine residues 413 and 424 were switched to glutamic acid), selected with puromycin for five days, and then treated with 500ng/mL doxycycline for 72 hours and examined by immunoblot analyses to examine expression of IRF6, lytic EBV proteins BZLF1, BRLF1, and LMP1, and the differentiation markers Involucrin, BLIMP1, KLF4, and ΔNp63α as shown. Tubulin served as a loading control. (TIF) [file ppat.1013236.s007.tif]

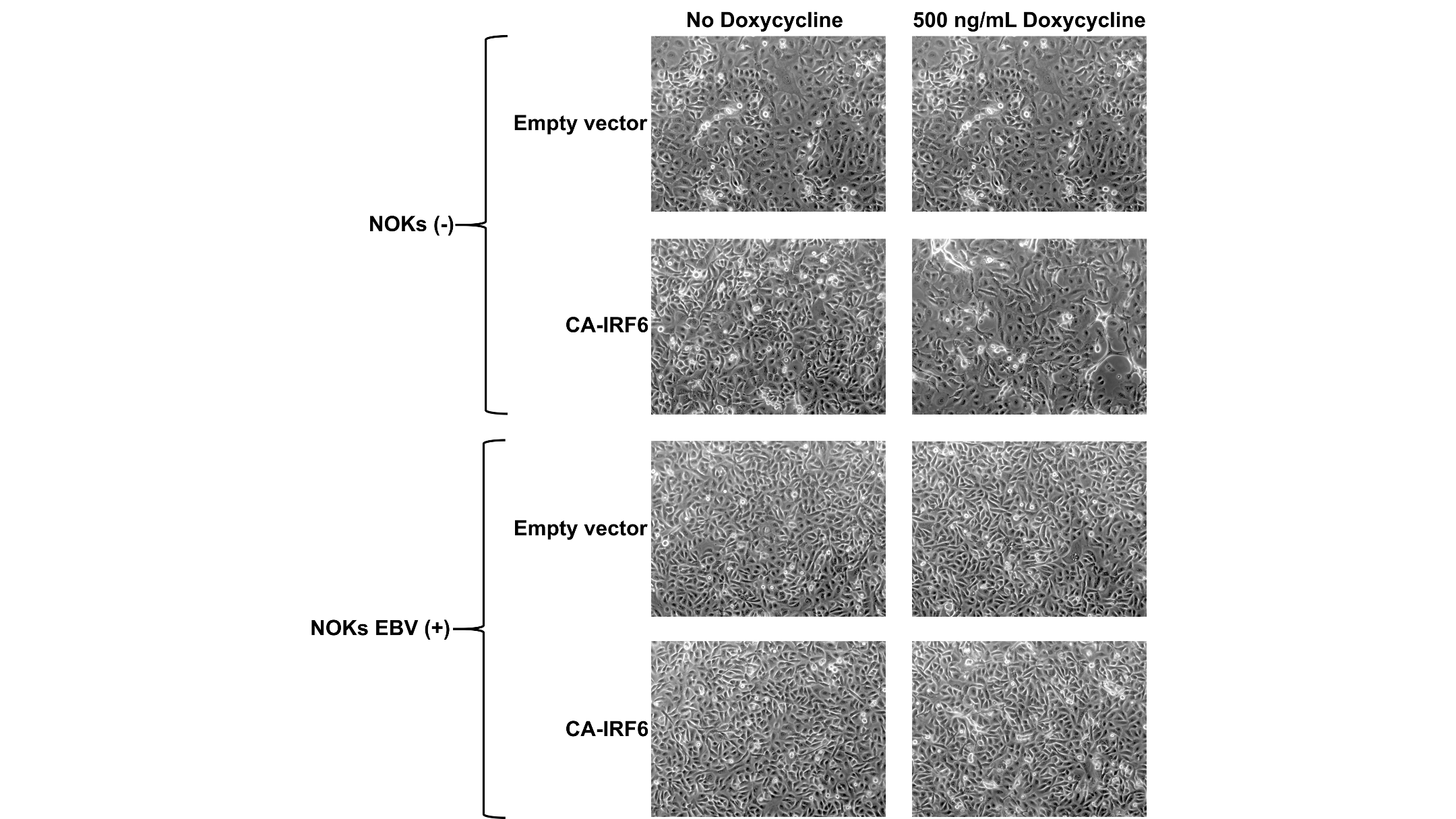

Supplement: S8 Fig — Akata type 1 strain EBV-infected NOKs were infected with a control vector or a lentivirus expressing a doxycycline inducible phospho-mimetic IRF6 mutant (CA-IRF6, in which serine residues 413 and 424 were switched to glutamic acid), then treated with 500ng/mL doxycycline for 72 hours and brightfield images were taken to examine cell health. (TIF) [file ppat.1013236.s008.tif]

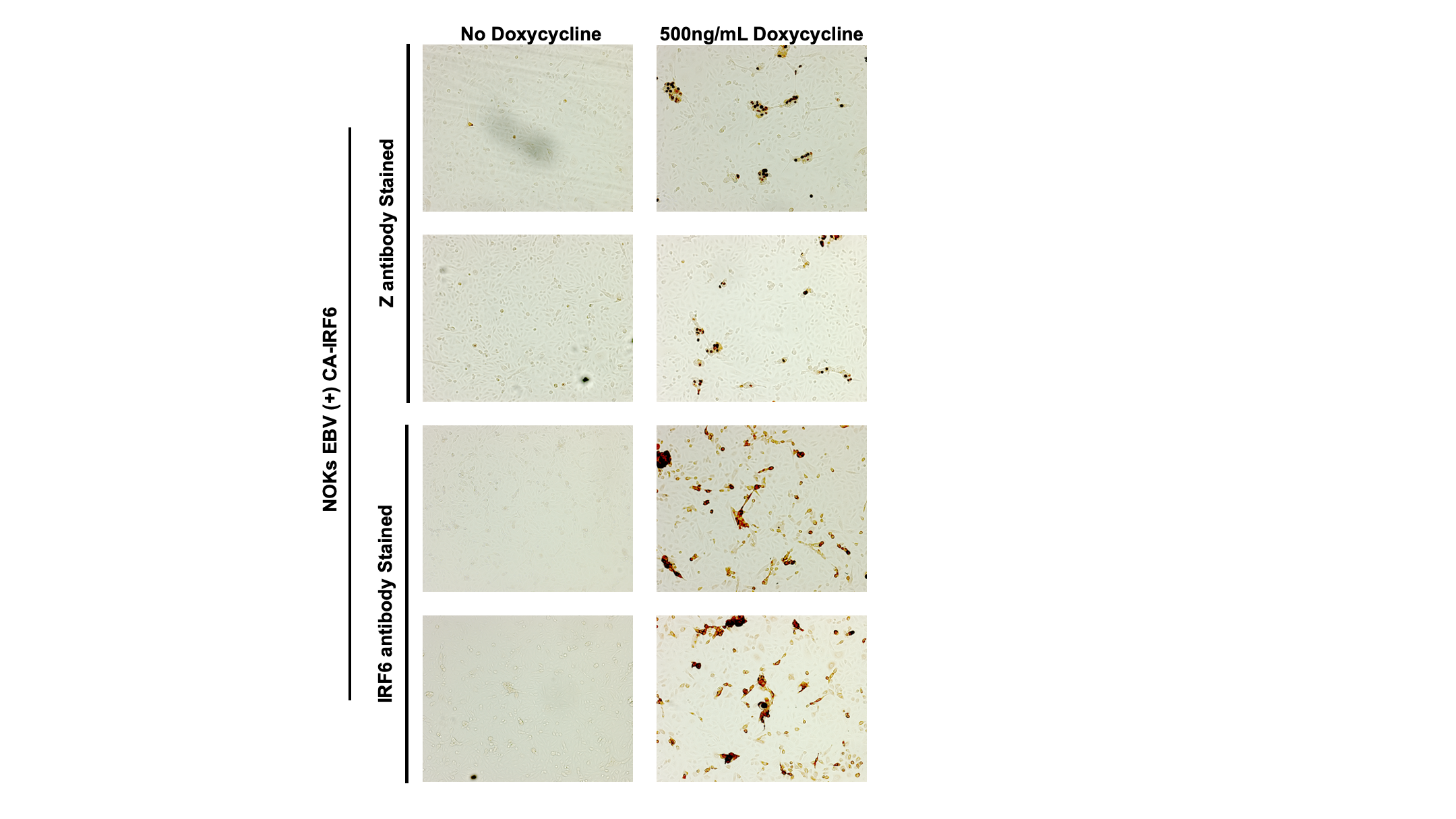

Supplement: S9 Fig — Akata type 1 EBV strain-infected NOKs were infected with a control vector or a lentivirus expressing a doxycycline inducible phospho-mimetic IRF6 mutant (CA-IRF6, in which serine residues 413 and 424 were switched to glutamic acid), plated on coverslips, then treated with 500ng/mL doxycycline for 72 hours. Cells were fixed using 3.7% formaldehyde, and stained using immunohistochemistry to detect IRF6 or BZLF1 expression and brightfield images were taken. Two separate fields stained with the anti-BZLF1 or anti-IRF6 antibodies from the same experiment are shown. (TIF) [file ppat.1013236.s009.tif]

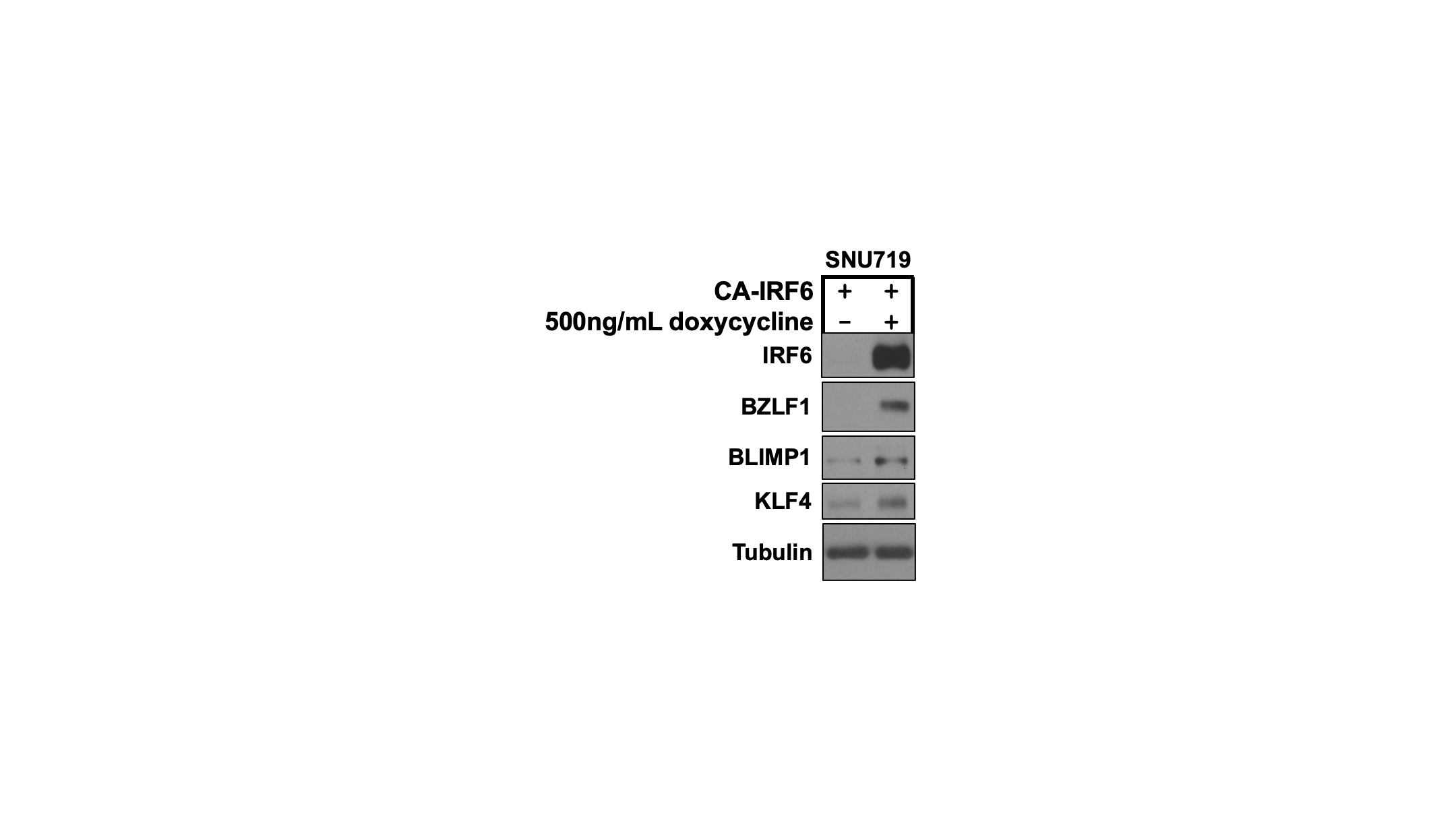

Supplement: S10 Fig — EBV-infected SNU719 cells were infected with a control vector or a lentivirus expressing a doxycycline inducible phospho-mimetic IRF6 mutant (CA-IRF6, in which serine residues 413 and 424 were switched to glutamic acid) then treated with 500ng/mL doxycycline for 72 hours and examined by immunoblot analyses to examine expression of IRF6, the lytic EBV protein BZLF1, and the epithelial cell differentiation markers BLIMP1 and KLF4 as shown. Tubulin served as a loading control. (TIF) [file ppat.1013236.s010.tif]

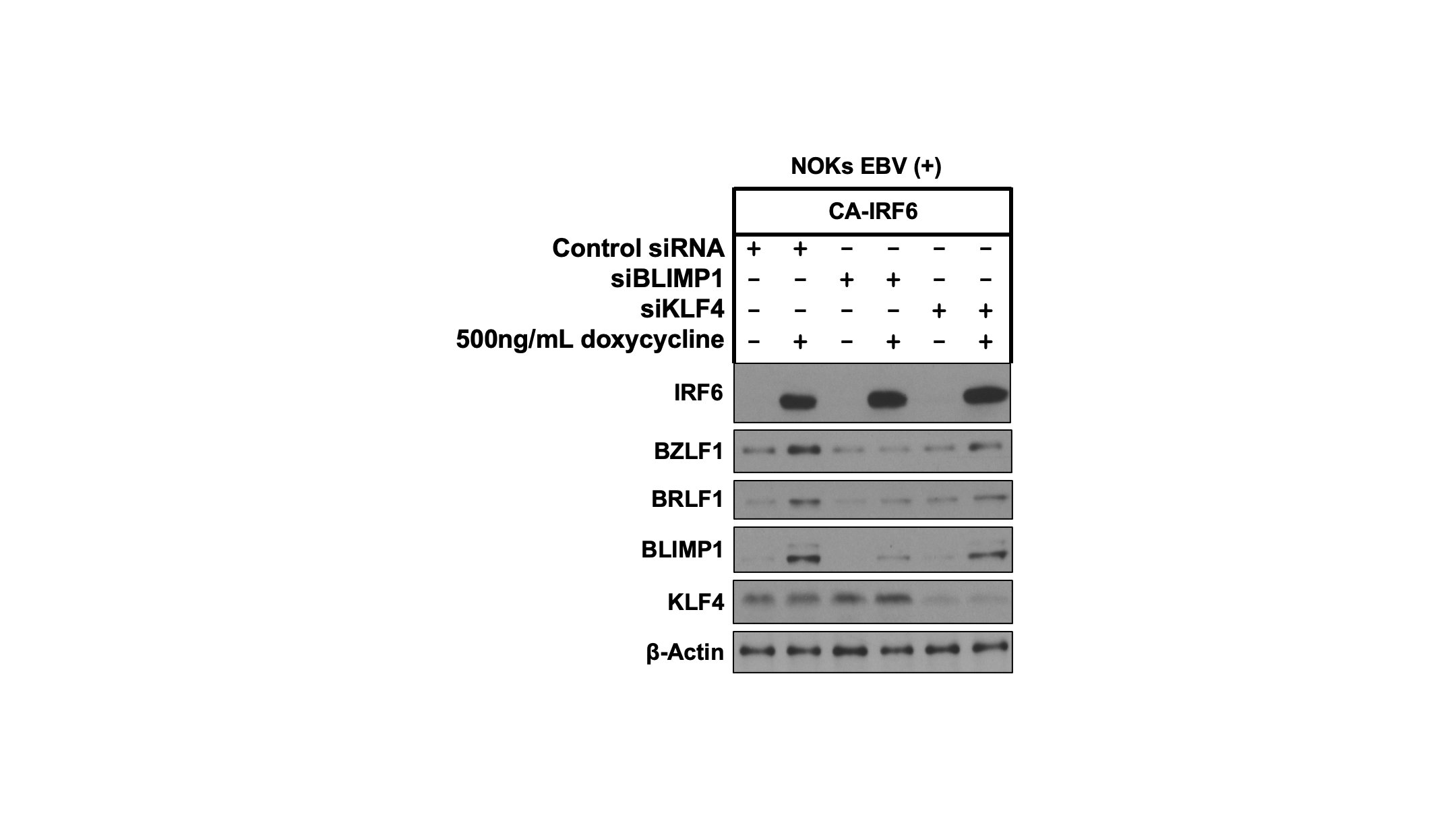

Supplement: S11 Fig — Akata type 1 EBV strain-infected NOKs were infected with a control vector or a lentivirus expressing a doxycycline inducible phospho-mimetic IRF6 mutant (CA-IRF6) and then transfected with control siRNA or siRNAs directed to KLF4 or BLIMP1 as indicated. 24h following transfection, cells were treated with 500ng/ml doxycycline for 72 hours and immunoblot analyses were performed to examine expression of CA-IRF6, BLIMP1, KLF4, BZLF1, and BRLF1 as shown. Actin serves as a loading control. Note that different control siRNA and IRF6 siRNA were used in this experiment compared to the experiments shown in Fig 8. (TIF) [file ppat.1013236.s011.tif]

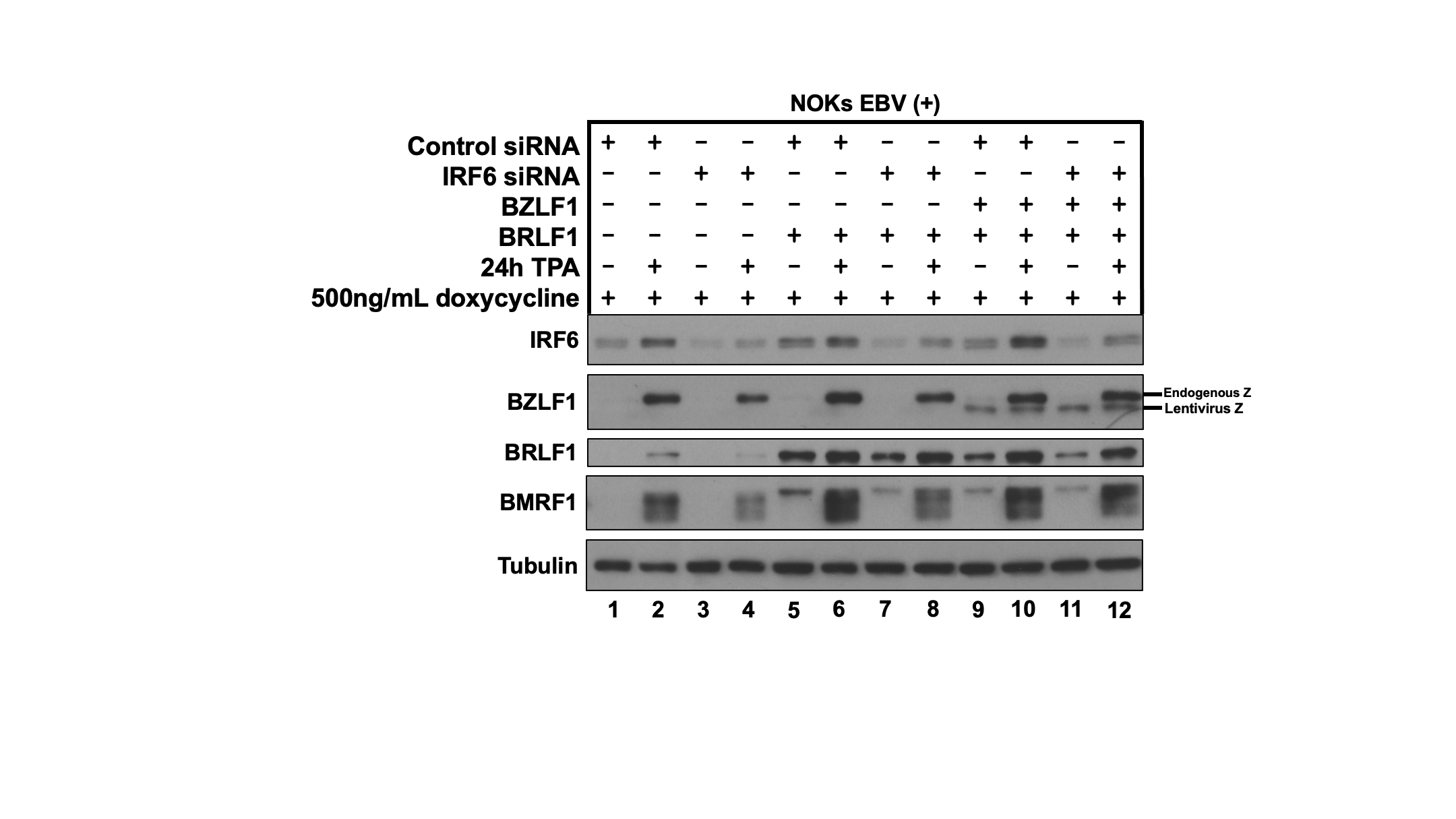

Supplement: S12 Fig — Akata type 1 EBV strain infected NOKs were stably infected with a control vector or a lentiviruses expressing doxycycline inducible BRLF1 alone or the combination of BRLF1 and BZLF1 EBV IE proteins, and then transfected with control siRNA or siRNAs directed to IRF6 as indicated. 24h following transfection, cells were treated with 500ng/ml doxycycline for 24 hours with and without TPA and immunoblot analyses were performed to examine expression of IRF6, BZLF1, BRLF1 and BMRF1 as shown. Tubulin serves as a loading control. The sizes of the BZLF1 protein (B95.8 strain) produced following BZLF1 lentivirus vector infection versus the endogenous (Akata strain) BZLF1 protein induced by TPA treatment are indicated. (TIF) [file ppat.1013236.s012.tif]
